# Coenzyme Q Biosynthesis: Evidence for a Substrate Access Channel in the FAD-Dependent Monooxygenase Coq6

**DOI:** 10.1371/journal.pcbi.1004690

**Published:** 2016-01-25

**Authors:** Alexandre Ismail, Vincent Leroux, Myriam Smadja, Lucie Gonzalez, Murielle Lombard, Fabien Pierrel, Caroline Mellot-Draznieks, Marc Fontecave

**Affiliations:** 1 Laboratoire de Chimie des Processus Biologiques, UMR 8229 CNRS, UPMC Univ Paris 06, Collège de France, Paris, France; 2 Sup’Biotech, IONIS Education Group, Villejuif, France; 3 Paris Sciences et Lettres (PSL*), Collège de France, Center for Interdisciplinary Research in Biology (CIRB), INSERM U1050, Paris, France; 4 Université Grenoble Alpes, Laboratoire Adaptation et Pathogénie des Microorganismes, Grenoble, France; 5 CNRS, Laboratoire Adaptation et Pathogénie des Microorganismes, Grenoble, France; Icahn School of Medicine at Mount Sinai, UNITED STATES

## Abstract

Coq6 is an enzyme involved in the biosynthesis of coenzyme Q, a polyisoprenylated benzoquinone lipid essential to the function of the mitochondrial respiratory chain. In the yeast *Saccharomyces cerevisiae*, this putative flavin-dependent monooxygenase is proposed to hydroxylate the benzene ring of coenzyme Q (ubiquinone) precursor at position C5. We show here through biochemical studies that Coq6 is a flavoprotein using FAD as a cofactor. Homology models of the Coq6-FAD complex are constructed and studied through molecular dynamics and substrate docking calculations of 3-hexaprenyl-4-hydroxyphenol (4-HP6), a bulky hydrophobic model substrate. We identify a putative access channel for Coq6 in a wild type model and propose *in silico* mutations positioned at its entrance capable of partially (G248R and L382E single mutations) or completely (a G248R-L382E double-mutation) blocking access to the channel for the substrate. Further *in vivo* assays support the computational predictions, thus explaining the decreased activities or inactivation of the mutated enzymes. This work provides the first detailed structural information of an important and highly conserved enzyme of ubiquinone biosynthesis.

## Author Summary

Coenzyme Q is an essential redox active lipid present in most living organisms and in tissues of multicellular eukaryotes, which acts as a key component in the mitochondrial respiratory chain. It consists of an aromatic head and a long hydrophobic isoprenyl chain and is synthesized by several enzymes within an obligate protein complex located at the inner mitochondrial membrane. Mutations in several human Q biosynthesis genes are responsible for a range of diseases that may be treated by oral supplementation of Q. Previous studies have established that Coq6, a predicted flavin-dependent monooxygenase, is responsible for the C5-hydroxylation reaction in the Q biosynthetis pathway, assisted by the ferredoxin Yah1 and ferredoxin reductase Arh1. We establish here that in *Saccharomyces cerevisiae* Coq6p uses FAD as a cofactor. We then use a computational approach to construct homology models of Coq6p, followed by MD calculations of the FAD-Coq6p complexes. These models are further used to get insight into the catalytic site structure and a general tunnel system in Coq6p. A specific tunnel is identified and mutations are proposed *in silico* in order to prohibit substrate access. The results of the *in vivo* study of the designed mutants substantiate our computational predictions.

## Introduction

Ubiquinone (coenzyme Q or Q) is an essential redox-active lipophilic molecule widely distributed from bacteria to mammals.[1,2,3] It is a key component of the ATP-producing mitochondrial aerobic respiratory chain in which it shuttles electrons from Complexes I and II to Complex III. Q also functions as a membrane-soluble antioxidant as well as a cofactor for the proton transport function of uncoupling proteins, and recently was further shown to act as a membrane stabilizing agent improving osmotic-stress tolerance in the bacterium *Escherichia coli*.[4] A fully-substituted benzoquinone ring mediates Q redox properties. This aromatic head is linked to a polyprenyl tail whose length varies between living organisms and which confers lipid solubility and ensures anchorage to cell membranes.[5] In *Saccharomyces cerevisiae*, it is formed by six isopentenyl units (ubiquinone is therefore designated as Q_6_), by 8 units in *E*. *coli* (Q_8_) and by 10 in humans (Q_10_). Q_10_ is well-known as a nutritional complement and a lipid-soluble antioxidant,[6] and is also used clinically to treat patients with coenzyme Q_10_ deficiencies.[7]

Ubiquinone biosynthesis corresponds to a highly-conserved pathway involving a large number of genes which are involved in the modification of a six-carbon ring derived from 4-hydroxybenzoate (4HB) into a quinone via attachment of the polyprenyl chain followed by hydroxylations, O-methylations and a C-methylation. [8] Despite seminal genetic and biochemical investigations on *S*. *cerevisiae* and *E*. *coli*,[9,10] this pathway is far from being completely understood. Indeed, only a small number of the biosynthetic proteins (referred to as the Coq/Ubi families) have been isolated and characterized biochemically and structurally. Q_6_ biosynthesis in *S*. *cerevisae* requires at least 12 such Coq proteins, namely Coq1p-Coq9p, Yah1p and Arh1p,[10,11] and the recently-identified Coq11p.[12] Several, if not all, of these proteins form a multi-protein complex associated with the mitochondrial inner membrane—the CoQ synthome—where the presence of every member is mandatory to properly produce Q_6_.[13,14] Such functional and structural interdependence of yeast Coq proteins complicates the elucidation of the whole synthesis mechanism, with single Coq gene knockouts accumulating only early pathway intermediates. In this context we recently initiated a long-term project aimed at providing new insights into the structure and function of Coq proteins in yeast. Coq6p from *S*. *cerevisiae* is of particular interest. It was initially proposed to be a flavin-dependent hydroxylase, but it was unclear whether it was involved in C1 or C5 hydroxylation.[10,15] However, genetic and biochemical studies in our group unambiguously established that it was specifically responsible for the C5 hydroxylation during Q_6_ synthesis in yeast, with the unexpected assistance of Yah1 and Arh1.[16,17] Primary Q_10_ deficiency is a rare recessive disorder associated with mitochondrial dysfunction, encephalomyopathy, ataxia, and cerebellar atrophy.[18,19] In addition, a recent clinical study of 13 patients identified 6 mutations on the homologous human COQ6 gene that cause steroid-resistant nephrotic syndrome with sensorineural deafness as a likely consequence of Q_10_ deficiency.[20]

We report here the first biochemical characterization of yeast Coq6p which establishes that Coq6p is a flavoprotein using FAD as a cofactor. In the absence of any crystallographic three-dimensional structure of this enzyme, we constructed several models using homology modeling followed by molecular dynamics simulations and analysis of the FAD-Coq6p model complexes. This allowed the identification of potential channels for access of lipophilic substrates to the active site. Using substrate docking calculations, a number of key residues were proposed for mutagenesis in order to block access to the substrate. Functional *in vivo* assays of these site-directed mutants are in agreement with our theoretical predictions, supporting the hypothesis that one particular Coq6p channel is involved in substrate binding. This work provides a molecular basis for further studies towards a deeper understanding of structure-function relationships with respect to Coq6p mutant dysfunctions.

## Methods

### Computational Methods

#### Manual homology modeling

The amino acid sequence of Coq6p was retrieved from the UniProt database (entry P53318).[21] Sequence homologs for which an X-ray-derived structure is available were found using the Phyre2 server.[22,23] The homologs with highest sequence homology (templates 4K22[24] and 4N9X) were downloaded from the Protein DataBank[25] and further submitted to a structure-based search using the DALI server,[26] returning 2X3N[27] as a structurally homologous template. Template-target alignment was performed using the DiscoveryStudio v3.1 [28] molecular modeling platform in parallel with the ClustalO alignment tool.[29] This process was facilitated by a reference multiple sequence alignment (MSA) of 119 Coq6p sequence homologs obtained by using the ConSurf server.[30,31] This MSA was used to adjust a target-template alignment of Coq6p with 2X3N, 4K22 and 4N9X templates, which was passed to MODELLER [32,33,34] as implemented in DiscoveryStudio. Since each template has sub-optimal or missing coordinates for some regions, we only retained template residues with reliable coordinates in this alignment. Effectively, we partitioned the Coq6p target sequence into distinct regions, each inheriting coordinates from a different template or construction procedure. Secondary structure assignments for the largest missing segments (the *si* loop and the Coq6 family insert) were generated using the Jpred server.[35]

#### Automated homology modeling

In order to investigate alternative methods for template selection, alignment, and model generation, we used the I-TASSER[36] and ROBETTA[37] automated servers using their default parameters. The I-TASSER and ROBETTA comparative modeling servers follow the same basic steps of template search, alignment, and model building. Both methods use sequence profile scoring methods to find and select the most appropriate templates. The methodological differences between I-TASSER, ROBETTA and MODELLER have been recently reviewed.[38]

#### Molecular mechanics setup

Protons were added using DiscoveryStudio at expected configurations for pH = 7. The AMBER99SB-ILDN[39] force field was used to provide molecular mechanics parameters to Coq6p. In order to build the three Coq6p-FAD complexes in a comparable fashion, FAD coordinates for all three models were imported from PHBH structure 1PBE[40] and minimized after binding site rotamer adjustment. Force field parameters for the FAD co-factor were those used by Sengupta *et al*.[41] The General Amber Force Field (GAFF)[42] with partial charges optimized at the 6-31G* level was employed for generating this data. The Coq6p-FAD complexes were put in a rhombic dodecahedron cell with walls 14 Å away, solvated with TIP3P[43] explicit water molecules with Na^+^ and Cl^-^ ions added at 0.157 M concentration as documented for the mitochondrial matrix.[44]

#### Molecular dynamics

All Coq6p-FAD models including the single (Coq6p-G248R and Coq6p-L382E) and double mutants (Coq6p-G248R-L382E) were subjected to molecular dynamics simulations in order to assess their structural stability and perform conformational sampling. All calculations were performed with GROMACS v4.6.5[45,46] on a dual Xeon workstation with an Nvidia Tesla C2075 GPU. A Particle-Mesh-Ewald electrostatics scheme was employed [47] with a shifted potential for long range interactions using the Verlet cut-off scheme[48] set at 10 Å. Models prepared as described above were first submitted to 300,000 steps of steepest descent minimization. Next, Coq6p heavy atoms were restrained and two 250 ps, 1 fs timestep, T = 300 K equilibration runs were performed. The first equilibration was performed in the NVT ensemble using the V-rescale Berendsen thermostat [49] with 0.1 ps relaxation time; the second equilibration was done in the NPT ensemble using the Parrinello-Rahman barostat[50] set at 1 atm. Constraints on Coq6p heavy atom positions were removed for production runs. Bonds between heavy atoms and hydrogens were constrained using LINCS.[51,52] All wild type models of Coq6p were subjected to 50 ns MD at a 2 fs time step, with Coq6p-FAD and the solvent coupled separately to the V-rescale Berendsen thermostat (NPT ensemble, 300 K, 0.1 ps relaxation time). Regarding MD calculations on Coq6p mutants, 20 ns runs were sufficient to yield unambiguous response regarding the impact of the mutation(s).

#### MD trajectory analysis

Basic analyses such as root mean square deviation (RMSDs) and secondary structure fluctuations on the MD trajectories generated by GROMACS were performed using the appropriate VMD plugins,[53] allowing both global and regional inspections. The variations of key interatomic distances were also monitored with VMD.

#### Analysis of reference conformations from the models

Coq6p evolutionary residue conservation was calculated with the ConSurf server from the Coq6p sequence, generating a multiple sequence alignment of 119 homologs, and mapped on Coq6p homology models. An analysis of potential substrate access channels on Coq6p was performed using the CAVER plugin[54] for PyMol [55]using a shell depth of 6 Å and a shell radius of 6 Å, with starting coordinates set in front of the FAD isoalloxazine. Tunnels leading to the catalytic site were characterized by their diameter at their narrowest points as measured between pairs of specific residues. Maxima in tunnel diameters were used as a criterion in selecting conformations from MD trajectories for substrate docking. Other tunnels were ignored.

#### A receptor-based scoring function

In the absence of experimental enzyme-substrate complexes for Coq6p or homologs, a receptor-based scoring function was developed to identify catalytically-plausible receptor conformations, defined as similar to that of PHBH (PDB entry 1PBE). The well-characterized binding mode of 4-HB (also called para-hydroxybenzoate, pHB) in this FAD-pHBH complex was used as a reference.[56] The scoring function is applied to every frame of the MD trajectory, providing a criterion for selecting enzyme conformations compatible with catalytic substrate binding to be used in subsequent docking. Residues forming H-bonds with 4-HB in pHBH, S212, P293 and T294, were identified and selected interatomic distances were used to create a 3D description of the catalytic site as bound to an aromatic substrate. The C4X FAD-atom was also used in the scoring function. The scoring function, S, was constructed as the sum of the differences between the catalytic site interatomic distances measured in the 1PBE crystal structure and those sampled from MD simulations of the pHBH-FAD complex:

$$S\;=\Sigma \left[{d\;}_{\text{ij}}\left(\text{Coq6p}\right){\text{ - d }}_{\text{ij}}\left(\text{1PBE}\right)\right]$$

A low score indicates a high structural similarity between the substrate-bound PHBH active site in the 1PBE structure and the substrate-free pHBH conformations sampled during MD.

#### Substrate docking on Coq6p-FAD models

AutoDock Tools 1.5.6[57] was used to prepare Coq6p-FAD and substrate model coordinate files for docking with AutoDock Vina.[58] Coq6p-FAD conformations were selected from MD trajectories using the criteria of channel diameter and the receptor-based scoring function previously mentioned. We used 3-hexaprenyl-4-hydroxyphenol (4-HP6) as a model substrate. Atoms were typed according to Vina’s standard table with Gasteiger charges. Only polar hydrogens were represented explicitly, identifying their parent atoms as possible hydrogen bond donors. Docking was performed without water molecules. Standard Autodock Vina scoring function terms and weights were used. Docking box dimensions were specified to restrict the receptor search volumes to the substrate access channels identified through the CAVER calculations. Exhaustiveness was increased to 96 runs to ensure good sampling. Substrate conformations were randomized at the beginning of each run of iterative local search. These initial conformations were randomly perturbed and then locally optimized with the BFGS algorithm. Resulting conformations were evaluated by Vina’s scoring function and selected according to the Metropolis criterion until convergence was obtained. Low-scoring conformations found during all runs were saved, clustered, and refined before being ranked again by Vina’s scoring function. The top 9 binding modes falling within a range of 3 kcal/mol according to the scoring function were retained for analysis.

### Experimental Methods

#### Strains and plasmids

BL21 (DE3) *E*. *coli* strains were purchased from Novagen. The pGro7 plasmid encoding the chaperones GroES and GroEL under the control of the inducible araB promoter was purchased from TaKaRa Bio, Inc. (Japan). The pMal-c2X plasmid overexpressing MBP (Maltose Binding Protein) fusion protein was purchased from New England Biolabs. The *coq6* gene from *S*. *cerevisiae* was optimized (Eurogentec) for expression in *E*. *coli* (S1 Fig) and then cloned in the pMAL-c2X expression vector between restriction sites BamHI and EcoRI, downstream the malE gene of *E*. *coli*.

The yeast plasmid pCM189 containing the *coq6* ORF under the control of its own promoter (pCoq6 plasmid) has been described [59] as well as the G248R mutant (pCoq6-G248R plasmid). Mutagenesis of *coq6* to introduce the L382E mutation was performed in two steps. First, two complementary mutagenic oligonucleotides, 3′L382Ecoq6 and 5′L382Ecoq6 (S1 Table), were each used in combination with an oligonucleotide complementary to the 5′ end (SacI coq6) and 3′ end (HindIII coq6) of *coq6*, respectively. The mutagenic oligonucleotides were designed such that leucine 382 is changed to a glutamate residue. The PCR reactions using oligonucleotides 3′L382Ecoq6 and SacI coq6 and oligonucleotides 5′L382Ecoq6 and HindIII coq6 yielded the DNA fragments *coq6-1* and *coq6-2*, respectively. The matrix used was pCoq6 or pCoq6-G248R. In the second step *coq6-1* and *coq6-2* were hybridized and used as templates for PCR amplification with the pair of oligonucleotides SacI coq6 / HindIII coq6. The resulting SacI-HindIII fragment was cloned into digested pCoq6, yielding the plasmids pCoq6-L382E and pCoq6-G248R-L382E. All constructs were verified by DNA sequencing.

#### Yeast strain, transformation and media

The yeast Δcoq6 strain (αW303ΔCOQ6-2) used for the complementation experiments with the Coq6 alleles has been described elsewhere.[60] DNA transformations were performed with the PEG-lithium acetate method as previously reported.[61] The cells were grown in YNB without pABA and folate (MP Biomedicals) supplemented with amino acids and nucleobases to cover the yeast auxotrophies except for uracil since the plasmids carry the URA3 gene. For solid media, 1.6% bacto-agar was added. 0.2% glucose and 2% galactose were used as carbon sources for pre-cultures. The pre-cultures were inoculated (100 fold dilution) into medium containing 2% lactate-2% glycerol for Q quantification or 2% galactose for the serial dilution phenotypic assay.

#### Expression and purification

Overexpression of the Coq6p-MBP fusion protein (Coq6p-MBP) was achieved by using BL21 (DE3) *E*. *coli* strain co-transformed with pMALc2x-Coq6p and pGro7. The cultures were grown in LB medium at 37°C in the presence of glucose (0.2%), ampicillin (100 μg/ml) and chloramphenicol (34 μg/ml) until they reached OD_600_ = 0.4 for induction of the chaperones GroES/EL with L-arabinose (2 mg/ml). When they reached OD_600_ = 0.8, cells were shifted to 18°C and the expression of Coq6p-MBP was induced by the addition of IPTG (150 μM). After overnight incubation at 18°C, cells were harvested by centrifugation. All subsequent operations were carried out at 4°C. The cell pellet was resuspended in 50 mM Tris-HCl, pH 7.5, 150 mM NaCl, 5% glycerol (v/v) in the presence of the protease inhibitor (cOmplete EDTA-free, Roche Life Science). After sonication, the lysate was submitted to ultracentrifugation at 180,000 x g for 90 min, at 4°C. The resulting supernatant was loaded onto an MBPTrap HP affinity column (GE Healthcare) pre-equilibrated with buffer 50 mM Tris-HCl, pH 7.5, 150 mM NaCl, 1 mM EDTA, and 5% glycerol (v/v). Coq6p-MBP was eluted with a maltose gradient (0–25 mM) and loaded onto a Superdex 200 (26/60) column (GE Healthcare) pre-equilibrated with buffer 50 mM Tris-HCl, pH 7.5, 150 mM NaCl, and 5% glycerol (v/v). The protein was concentrated with spin concentrators (Vivaspin 30-kDa cutoff) up to 10 mg/ml in the same buffer, frozen in liquid nitrogen then stored at -80°C.

#### Biochemical characterization

The molecular mass of Coq6p-MBP was determined using a gel filtration column (Superdex 200 10/300 GL) calibrated with standards of known molecular mass (Bio-Rad). The protein concentration of Coq6p-MBP was determined with the Bradford assay.[62] The nature and occupancy of the Coq6p-MBP cofactor were determined using HPLC and UV-visible spectrophotometry. One hundred microliters of purified Coq6p-MBP (30 μM) was thermally denatured (100°C for 10 min). After centrifugation (10 min at 13,000 x *g*), the supernatant was analyzed on a Hypersil Gold C-18 analytical column (1.9 μm, 2.1 x 50 mm, Thermo Scientific) which was run with methanol/ammonium acetate 5 mM (pH 6.5) (10/90). The flavin cofactor was identified by comparing retention times and visible absorption spectra with standards (FAD, FMN). The flavin concentration of the solution was determined by UV-visible spectrophotometry, using the known extinction coefficient of free FAD (ε_450nm_ = 11300 mM^-1^.cm^-1^), and the flavin content of the protein was then calculated using the protein concentration determined above. Absorption spectra were recorded on a Cary 60 UV-Vis spectrophotometer apparatus (Agilent). Proteolytic cleavage of the MBP tag was performed by incubating the fusion protein Coq6p-MBP (1mg/ml) in 20 mM Tris-HCl, 100 mM NaCl, 2 mM CaCl_2_, glycerol 5%, pH 8.0, with Factor Xa (New England Biolabs, 1/50, enzyme/substrate w/w) at 23°C overnight. The reaction was stopped with 2 μM of the Factor Xa inhibitor Dansyl-Glu-Gly-Arg-chloromethyl ketone (Merck Millipore), and the resulting cleaved proteins were analyzed by SDS PAGE. The ability of NADH and NADPH to reduce the FAD cofactor of Coq6p-MBP was monitored by UV-visible absorption spectroscopy. A 10 μM solution (100 μl) of Coq6p-MBP was titrated with successive addition of 5 μl aliquots of 1 mM reductant. NADH oxidase activity was tested spectrophotometrically by monitoring the decrease in absorption of NADH at 340 nm (ɛ_340_ = 6.22 mM^-1^cm^-1^) at 25°C. The assay mixture contained 0.2 mM NADH in 50 mM sodium phosphate buffer, 5% glycerol (v/v), pH 7.4. The reaction was initiated by the addition of Coq6p-MBP (0.05–2 μM). Enzymatic assays for substrate hydroxylation by Coq6p-MBP were performed by monitoring NADH/NADPH consumption at 340 nm, and the reaction mixtures from these assays were analyzed by HPLC. A typical reaction mixture (1 ml final) contained 1 mM NADH or NADPH, 1 mM substrate, and 1–10 μM Coq6p-MBP in 20 mM Tris buffer, 100 mM NaCl, 1 mM EDTA, pH 7.5, and was incubated for 2 hours at 25°C. The protein was then precipitated with one volume of acetonitrile:ethanol (10:1) at 4°C, centrifuged for 10 min to remove precipitated proteins. The supernatant (500 μl) was then injected into an Atlantis Prep T3 column (5 μm, 10 x 250 mm, Waters), eluted by a gradient of acetonitrile (0–100%) at a flow rate of 0.5 ml/min and organic compounds were monitored at 254 nm.

#### Q extraction and quantification

Cells from cultures at OD_600_~2 were collected by centrifugation, washed once with ice cold water and their wet weight was determined in pre-weighted Eppendorf tubes before freezing at -20°C. Glass beads (100 μL), 50 μL KCl 0.15 M, a Q_4_ solution (4 μM in methanol, 2 μL/mg of wet weight) and 0.6 mL methanol were added to cell pellets (10–30 mg wet weight). Tubes were vortexed for 10 min. Neutral lipids were extracted by adding 0.4 mL petroleum ether (40°-60° boiling range) and by vortexing for 3 min. The phases were separated by centrifugation (3 min, 1000 g, room temperature). The upper petroleum ether layer was transferred to a fresh tube. Petroleum ether (0.4 mL) was added to the glass beads and methanol–containing tube, and the extraction was repeated once more. The petroleum ether layers were combined and dried under argon. The lipids were resuspended in 100 μL methanol, and aliquots were analyzed by reversed-phase high-pressure liquid chromatography (HPLC) on a Dionex U3000 system equipped with a C18 column (Betabasic-18, 5 μm, 4.6 x 150 mm, Thermo Scientific) at a flow rate of 1 mL/min. The mobile phase was 5% isopropanol, 20% acetonitrile, 75% of (98% methanol and 2% 1M ammonium acetate in water). Quinones were quantified with an ESA Coulochem III electrochemical detector (ECD) and a 5011A analytical cell (E1, -550 mV; E2, 550 mV). Hydroquinones present in samples were oxidized with a precolumn 5020 guard cell set in oxidizing mode (E, +600 mV). The standard Q_4_ solution was injected in the same conditions to generate a standard curve which was used to correct for sample loss during the organic extraction (on the basis of the recovery of the Q_4_ internal standard) and to quantify Q_6_.

## Results

### Purification and Characterization of Coq6p

**Fig 1:**
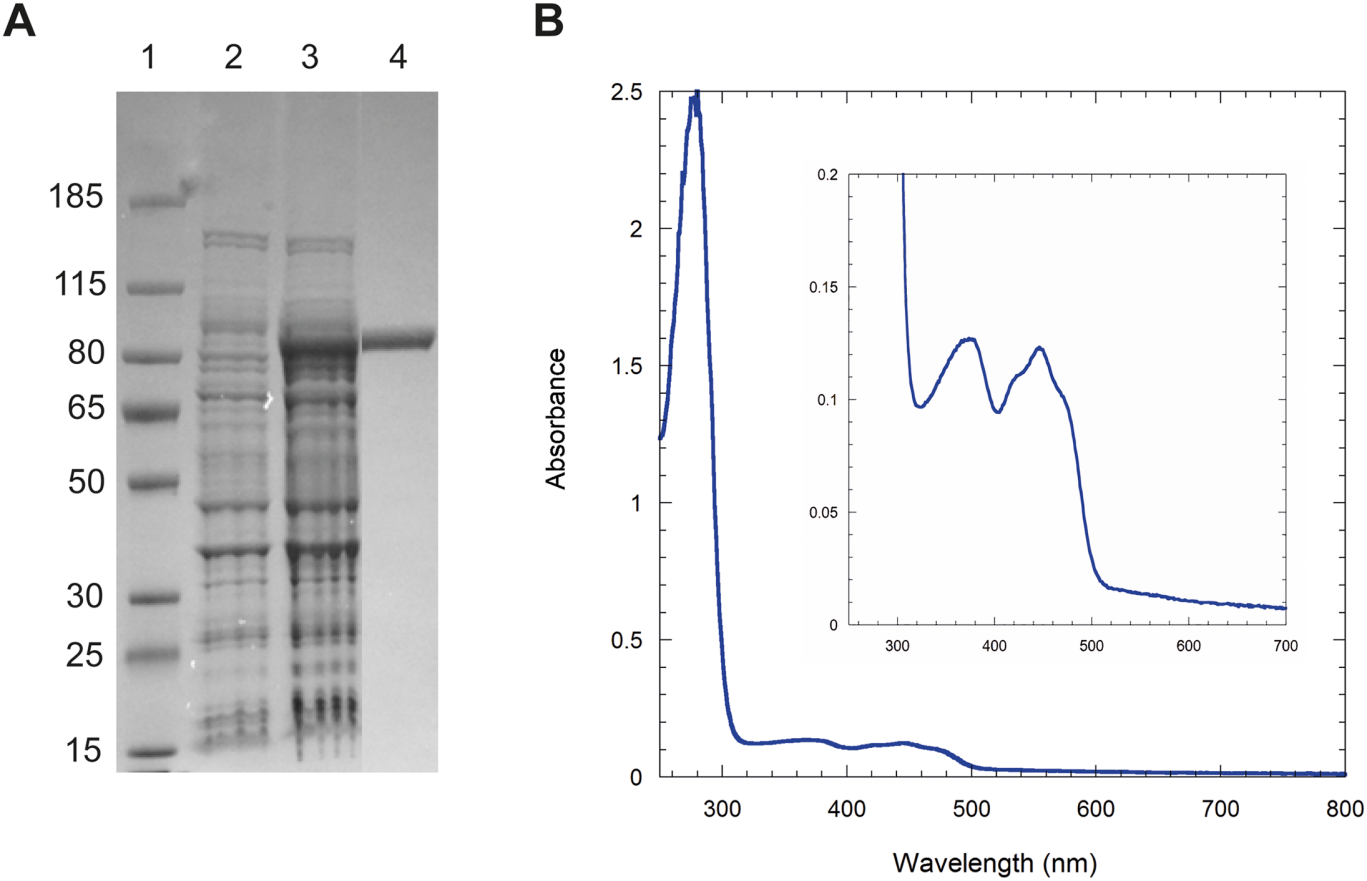
Coq6p biochemical characterization. A) SDS PAGE (4–12% Bis-Tris, MOPS). Lane 1, Molecular Weights (kDa); lane 2, BL21 DE3 cells transformed with pMALc2X-Coq6 before IPTG induction; lane 3, cells after 20 hr-IPTG induction at 18°C; lane 4, Coq6p-MBP (96.3 kDa) after a two-step purification (MBP trap, Superdex200 26/60). B) UV-visible spectrum of purified Coq6p-MBP, 1.20 mg/ml.

Coq6p was overexpressed in the presence of GroES and GroEL chaperones, and purified as an N-terminal MBP-tagged protein (named Coq6p-MBP) with a molecular mass of 96 kDa (Fig 1A). Gel filtration experiments indicated that the protein formed tetramers and high molecular weight oligomers that could be further separated. The yellow color of the tetrameric Coq6p-MBP and its UV-visible spectrum with characteristic absorption bands at 370 nm and 446 nm (Fig 1B) show that it contains a flavin cofactor. The flavin extracted from Coq6p-MBP was identified as flavin adenine dinucleotide (FAD) based on its co-elution on HPLC with commercial standard (S2 Fig). FAD occupancy was estimated at 85% per monomer in the tetrameric Coq6p-MBP by UV-vis spectroscopic analysis. FAD occupancy was estimated at 85% per monomer in the tetrameric Coq6p-MBP by UV-vis spectroscopic analysis and Bradford assay. Because the large size of the MBP tag (45 kDa) may impede protein function, removal of this tag from Coq6p-MBP was attempted using enzymatic cleavage by Factor Xa. However it resulted in protein aggregation of cleaved Coq6p. The reduction of the FAD cofactor was attempted with different reductants (NADH and NADPH) in anaerobic conditions and monitored by UV-visible spectroscopy. The FAD cofactor of Coq6p-MBP could be reduced neither by NADH nor NADPH. No NADH oxidase activity of Coq6p-MBP was detected in aerobic conditions. We examined the ability of Coq6p-MBP to function as a flavin monooxygenase that utilizes dioxygen to catalyze the hydroxylation of various water-soluble aromatic compounds as models of the two potential substrates, 3-hexaprenyl-4-hydroxyphenol (4HP6) and 3-hexaprenyl-4-hydroxybenzoate (4-HB6). However, none of the tested molecules (4-hydroxybenzoic acid, 3-methyl 4-hydroxybenzoic acid, 2-methyl 4-hydroxyphenol, 2-methylphenol) were found to be substrates. Several hypotheses can explain this lack of activity: the presence of the MBP tag can affect the enzymatic activity of Coq6p-MBP; potential protein partners may be required for Coq6p to be enzymatically active; the positioning of Coq6p in the lipid membrane may also affect its biological activity, folding and binding with its substrate.[63]

### Summary of the Modeling Strategy

The modeling strategy is divided into four successive sections, whose results are presented and analyzed separately. First, we created homology models of Coq6p. We used two distinct approaches: one where template selection, template-target alignment, and model refinement steps are done with maximal user intervention using MODELLER[32–34] and the other using two highly ranked servers, ROBETTA and I-TASSER,[64,65] that fully automate these processes. At this stage, the FAD co-factor was introduced after model construction, as detailed in Methods. Second, the three Coq6p-FAD models were subjected to molecular dynamics simulations. The resulting trajectories were analyzed on the basis of substrate accessibility to the catalytic site by preliminary docking calculations on a model substrate, 4-HP6. At the end of this process, one of the models was selected for further analysis. Third, detailed substrate docking in the wild type enzyme was performed. Representative enzyme conformations were extracted using a receptor-based scoring function designed to recognize catalytic site conformations likely to be compatible with catalysis. Finally, mutations were rationally designed on the basis of *in silico* results and experimentally tested by *in vivo* assays.

### Template Selection and Target-Template Alignments

The search for proteins of known structures presenting sequence homology with Coq6p consistently identified FAD-dependent monooxygenases. Such proteins constitute appropriate templates for homology modeling and their crystal structures are identified as follows in the PDB database: 4N9X, 4K22,[24] 2X3N,[27] and 1PBE.[40] Sequence alignment of Coq6p with these templates reveals low sequence identities (28.32%, 27.30%, 20.30%, 18.51%, respectively, see S3 Fig).

In spite of regions with unsolved coordinates in several of the templates, these templates exhibit similar overall tertiary structures with a Rossmann-like β/α/β fold forming an FAD-binding domain and a large beta sheet forming a substrate binding domain. Structural comparison shows that the core secondary structure elements are overlapping (S4 Fig).

**Fig 2:**
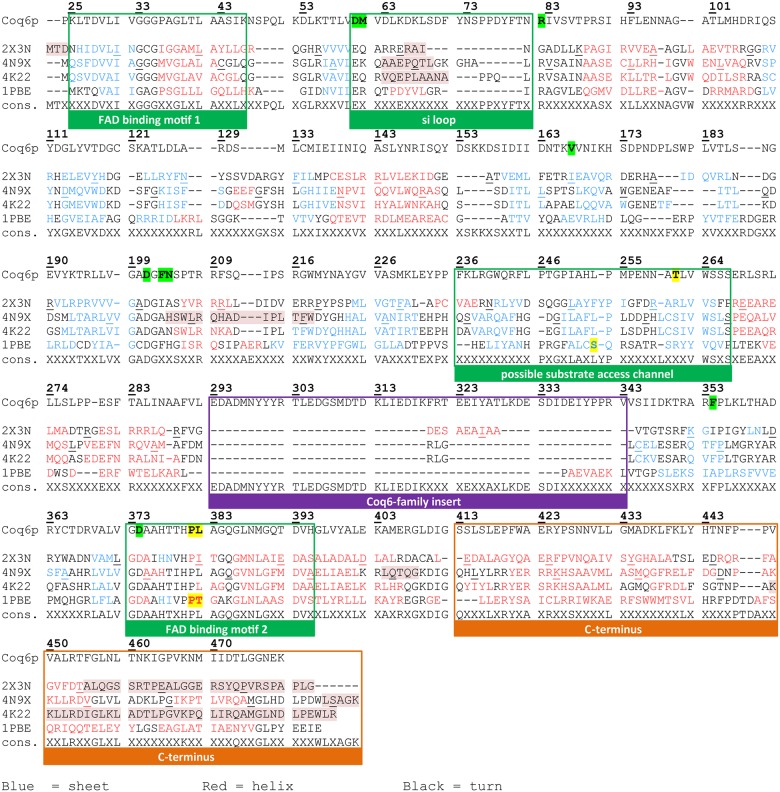
Sequence alignment of Coq6p with templates used by the different homology modeling approaches. The structural annotations are derived from studies of pHBH, the holotype for this class of enzymes. The templates are referred to by their PDB codes. 2X3N is a putative quinolone monooxygenase from *Pseudomonas aeruginosa* (1.75 Å resolution); 4N9X, a putative ubiquinone biosynthesis monooxygenase from *Erwinia carotovora* (2.5 Å resolution); 4K22, a ubiquinone biosynthesis hydroxylase from *E*. *coli* (2 Å resolution); 1PBE, the para-hydroxybenzoate hydroxylase from *Pseudomonas fluorescens*. Secondary structure as calculated by DSSP is indicated by text color: beta strands are blue, alpha helices are red, and turns are in black. Residues missing from crystal structures have background highlighted in red. Residues selected for building the active-site geometry descriptor and scoring function are highlighted in yellow. FAD binding residues are highlighted in green.

If one assumes that such structural features also translate to Coq6p and that they are correlated to Coq6p function, then an approach based on homology modeling is relevant. In order to resolve alignment ambiguities among these low homology sequences, the pairwise alignments of Coq6p with 4K22,[24] 4N9X, 2X3N,[27] and 1PBE[40] were manually curated by cross-referencing them against a multiple sequence alignment of 119 Coq6p homologues (S5 Fig) as well as by considering the 3D structure of each template. The resulting target-template pairwise alignments were then collated to build a structurally annotated alignment for Coq6p shown in Fig 2. Compared to the templates, it is apparent that Coq6p contains a long additional sequence designated the Coq6 family insert (293–343), with the insert of Coq6p *S*. *cerevisiae* being among the longest in the Coq6 family (S5 Fig). Target-template alignments for each of the three modeling protocols are presented in S6 Fig. ROBETTA automatically chose a single template, 1PBE. I-TASSER proposed models from 4N9X followed by 2X3N; we rather chose the 2X3N-derived model on the basis that it is co-crystallized with FAD, ensuring a reliable initial conformation of the FAD binding domain, unlike 4N9X. Based on structure-function knowledge, the alignment passed to MODELLER combined 3 templates: 2X3N for most of Coq6p N-terminal region (residues 1–426), 4N9X for the C-terminal region not present in 2X3N (residues 427–479), and 4K224 for a small 6-residue segment of the beta-sheet domain (residues 352–357). In this manual homology modeling step, we superposed both helices 12 of 2X3N and 4N9X to determine the relative orientation between the two templates, selecting P426 as the final residue of the 2X3N template and S427 as the starting residue of the 4N9X template. Regarding the small template sequence of 4K22, we similarly superposed the beta-strand of 4K22 and 2X3N templates to allow the modeling of this region in Coq6p_MODELLER. This small region of 4K22 was selected as the template one (rather than 2X3N) in order to allow the FAD to undergo in and out movements during subsequent MD runs, which do not occur using 2X3N for this region.

### Homology Models

**Fig 3:**
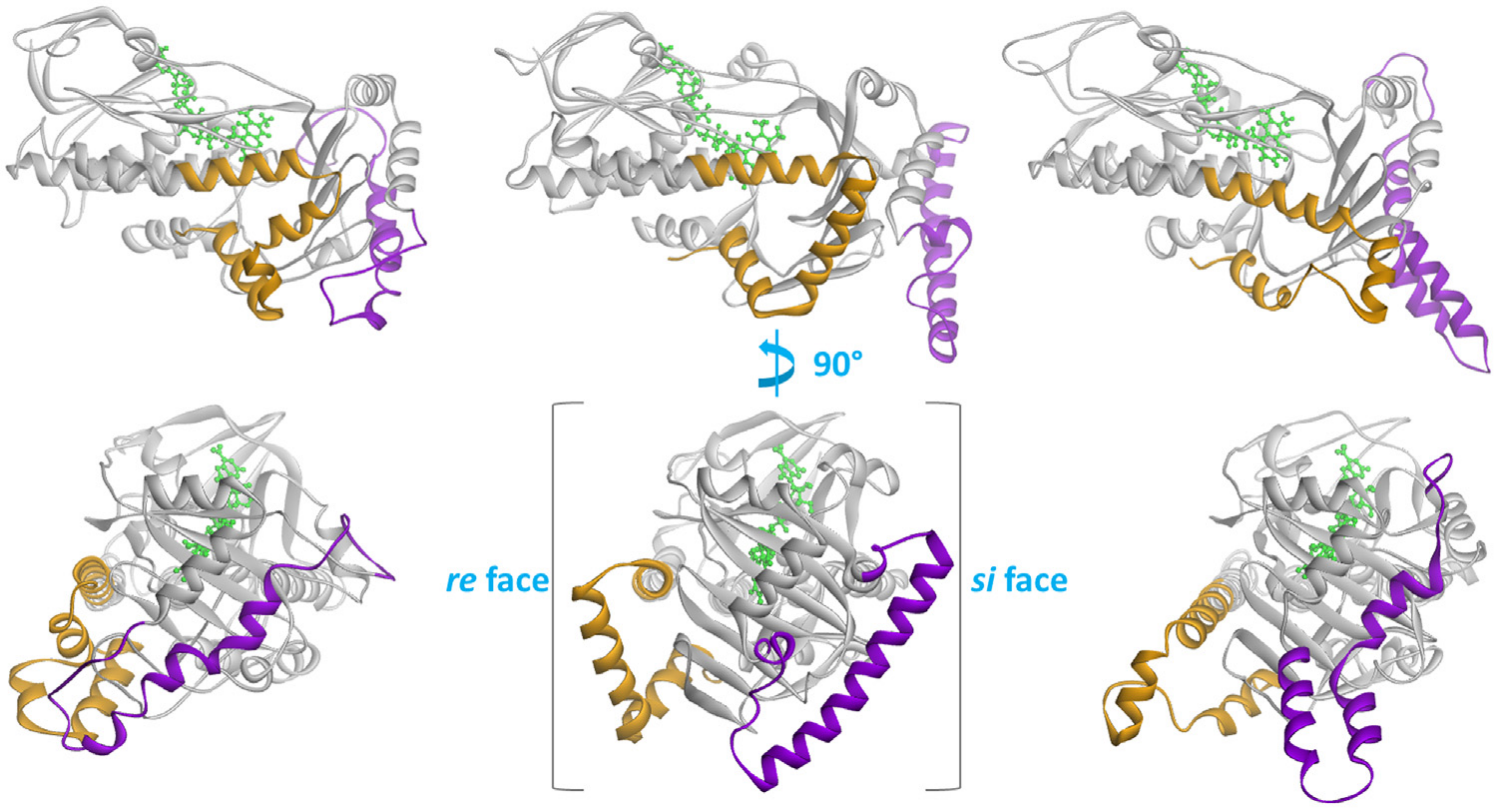
Comparison of the three Coq6p homology models. The Coq6p enzyme secondary structure is shown in cartoon, while the FAD cofactor is shown in green stick. Structurally divergent Coq6p secondary structure elements are highlighted in orange (the C-terminus) and purple (the Coq6-family insert). From left to right: Coq6p_I-TASSER model, Coq6p_ROBETTA model, and the Coq6p_MODELLER model. The isoalloxazine ring plane of the FAD co-factor has two faces, named *si* and *re*, with the relevant stereocenter for naming the faces being the C2’ carbon of the ribityl chain. Each face of the FAD’s ring is used here to designate the half of the Coq6p enzyme extending from the *si* and *re* faces of the FAD binding pocket to the edge of the protein. The top row shows the models from the *re* face, while the bottom row is rotated 90 degrees about the vertical to show the exterior of the beta sheet domain, which bears the insert.

Fig 3 highlights structural differences between the three homology models, Coq6p_I-TASSER, Coq6p_ROBETTA and the Coq6p_MODELLER model (based on a manually curated alignment of rationally selected templates), prior to MD. It is interesting that in all monooxygenase structures from the PDB that are *bound* to FAD, the FAD binding pocket is always constituted of a small ribityl binding loop (the GDAxH loop) structured as a α-helix, suggesting that a catalytically competent Coq6p structure must also have its ribityl binding-loop in a α-helix. All three models are derived from templates with a GDAxH loop compatible with FAD binding (see S1 Text), with the *si* loop (for which the templates are incompletely resolved) consistently reconstructed as a helix. Two major structural elements discriminate the models: the C-terminus and the 51-residue Coq6p-family insert. The former strongly depends on template choice, with the ROBETTA and the MODELLER models using 1PBE and 4N9X respectively, while the I-TASSER model does not rely on any template in this region, since it is not present in 2X3N. The Coq6 family insert is not present in any of the templates; therefore its final conformation in the models solely results from methodological differences for modeling *ab initio* regions. It is however consistently predicted to have a helix-turn-helix secondary structure. The insert is systematically localized to the exterior face of the beta-sheet domain, with no residues proximal to the active site. Since all flavin-dependent monooxygenases with known structure show catalytic activity without a similar structural element, the Coq6p insert is unlikely to be required for catalysis. However, its conservation among the Coq6 family (S5 Fig) and its high proportion of polar and charged residues suggest that it may be essential for integration of this enzyme into the obligate CoQ biosynthesis complex through ionic protein-protein interactions. Overall, the three homology models provide us with three significantly different starting models for further MD runs. Taking the Coq6p_MODELLER model as a reference, the Coq6p_ROBETTA and Coq6p_ITASSER models exhibit RMSDs of 6,53 Å and 6,39 Å respectively, which reflect the different structures of the insertion and of the C-ter region in the three models (see Fig 3). Additional RMSDs are given in S2 Table.

### Molecular Dynamics Analysis

**Fig 4:**
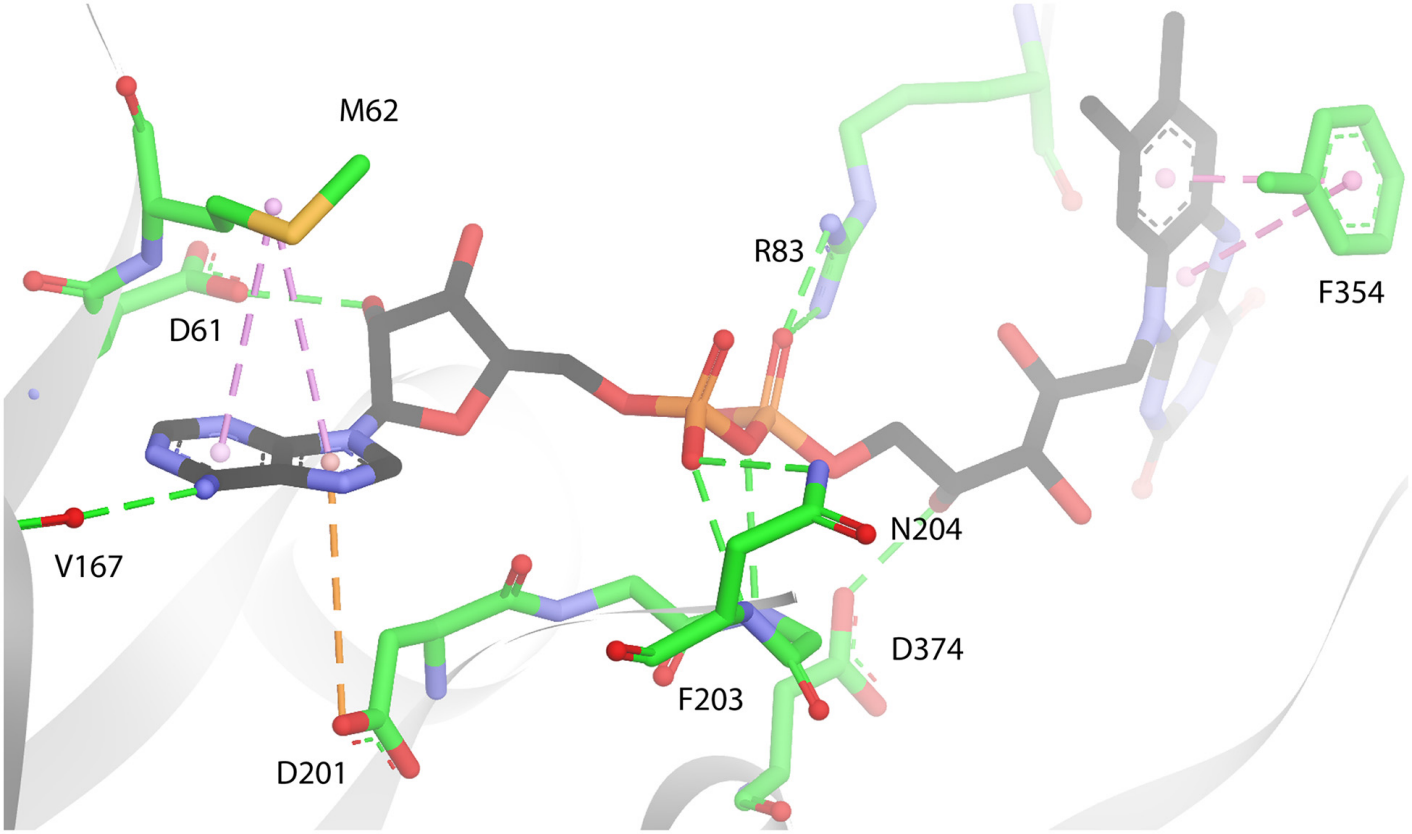
Model of FAD bound to Coq6p, as shown in the Coq6p_MODELLER model. This pose is from a FAD bound conformation selected from MD simulations (C: black, N: blue,O: red, P: orange). Hydrogen bonds are in green dashed lines, and π-type interactions with FAD’s aromatic cycles in purple dashed lines (centre of mass are shown as clear pink spheres). Residues are in sticks (C: green, S: yellow, O: red, N: blue). The adenine ring’s N3 nitrogen is H-bonded to V167. The adenine aromatic system can also form π-sulfur interactions with M62 and π-anion interactions with D201. The ribose hydroxyl is H-bonded to D61. The pyrophosphate is H-bonded to the side chains of R83 and N204, as well as the backbone nitrogen of F203 (side chain omitted for clarity). The ribityl chain is H-bonded to the side-chain of D374, the D of the GDAxH motif. The isoalloxazine ring can form π − π interactions with the F354 side chain (backbone omitted for clarity). These residues are highly conserved in the Coq6 family (S5 Fig; highlighted in green in Fig 2), with the exception of M62 and F203.

All three Coq6p-FAD homology models were subjected to the same molecular dynamics (MD) protocol. The aim of this process is first to investigate the structural stability of the models and perform conformational sampling of each model, which is important for subsequent substrate docking. All models appeared structurally-stable through 50 ns MD runs, all having converged to a conformational plateau in which RMSD fluctuations are as expected for proteins of this size simulated in physiological conditions (S7 Fig), all having stable secondary structures (S8–S10 Figs). This suggests that approximations and assumptions made during the modeling process (related to the *ab initio* modeling procedure for sections with no templates, or the manual addition of FAD post Coq6p model generation) did not prevent any of the models to be close to a low-energy state. Conformations of the three Coq6p models after MD are illustrated in S11 Fig. It is noted that the regions identified as most important for Coq6p function, such as the FAD-binding domain helices and the β−sheet domain, appear more stable structurally than the rest of the protein and especially the Coq6p insert and C-terminal region (as observed from their RMSDs in S7 Fig). The FAD binding mode as exemplified in Fig 4 was stable in the three homology models over the course of 50 ns MD calculations.

### Catalytic Site Identification

**Fig 5:**
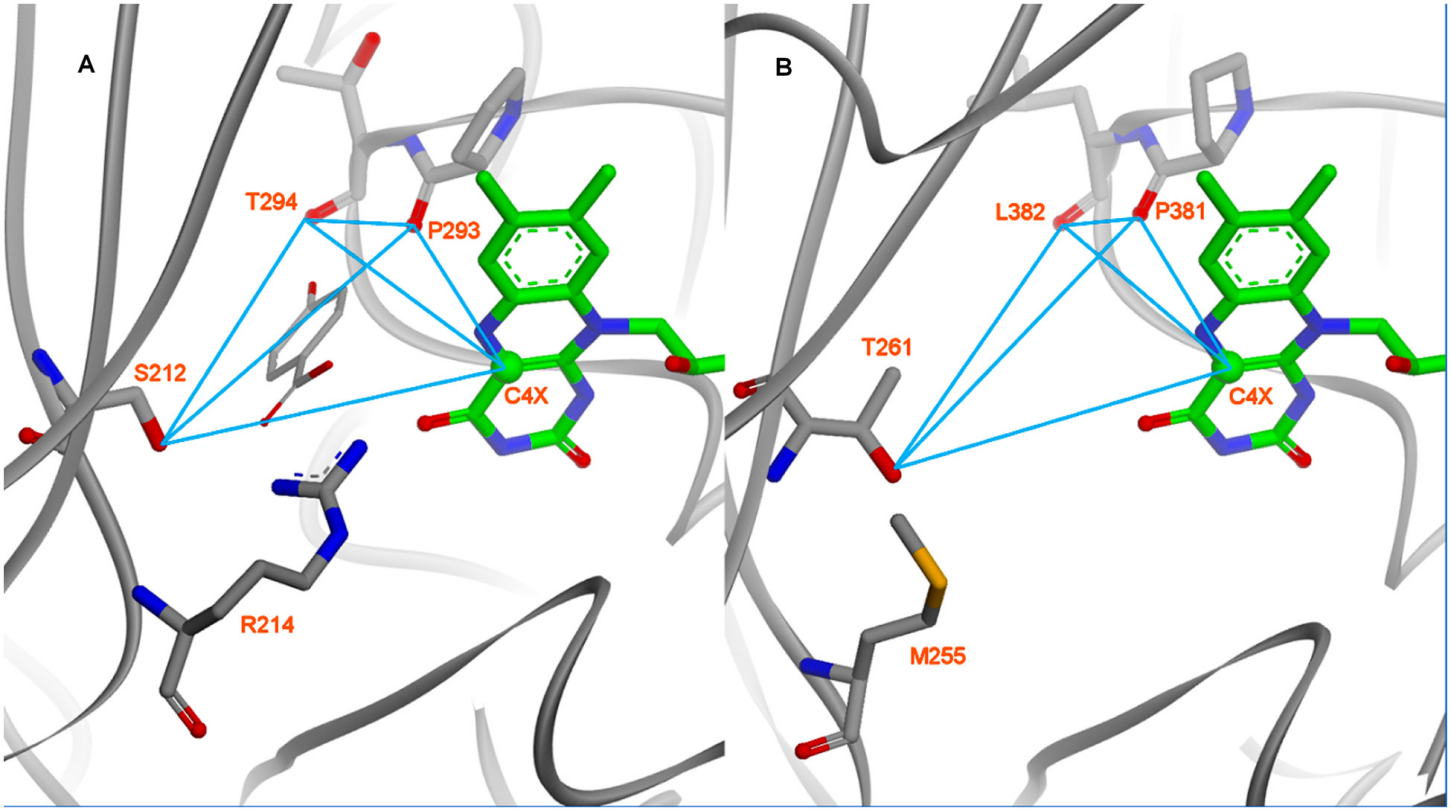
Comparison of pHBH’s active site crystal structure with the same region in the Coq6p model (here, the Coq6p_MODELLER model). The active site interatomic distances used for the scoring function-based substrate docking are shown as blue lines (see Methods). A): The co-crystal structure of para-hydroxybenzoate in pHBH (1PBE) provides coordinates of a structurally and functionally homologous enzyme-substrate-co-factor complex. In order to capture the active-site geometry compatible with substrate binding, a receptor-based geometric descriptor is derived from the enzyme’s hydrogen bonding partners with the substrate. B): Residues and atoms used in the geometric descriptor derived for pHBH are mapped onto homologous residues and atoms in the Coq6p catalytic site.

As strongly suggested by the position of FAD in both 1PBE and Coq6p models, the most-plausible location of the Coq6p catalytic site is a buried volume immediately facing the isoalloxazine ring, about 14 Å away from the protein surface. Focusing on this specific region (Fig 5), key residues were identified in both the 1PBE crystal structure, in which they contact the substrate 4-HB (S212, R214, P293 and T294, Fig 5A), and in Coq6p (T261, M255, P381 and L382, Fig 5B). These residues were used to build the receptor-based scoring-function with the exception of M255 to identify catalytically plausible poses. Three of these residues (T261, P381 and L382) are very conserved among Coq6 eukaryotic sequences (S5 Fig). They provide the same arrangement of 1 H-bond donor (PHBH: S212/Oγ; Coq6p: T261/Oγ) and 2 H-bond acceptors (P293,T294/O; P381-L382/O). Coq6p M255 cannot provide similar interactions to pHBH R214 (H-bonding to the 4-HB carboxyl group) although it is located at the same position, and hence was not included in the scoring-function. The 4-HB position in pHBH also gives us a diagnostic distance (4.32 Å) between the C4X FAD-atom which bears the peroxo group of the reactive FAD-OOH intermediate and the substrate target carbon to be hydroxylated.[40] The FAD C4X atom position in Coq6p relative to T261, P381 and L382 is also well predicted when compared to that in 1PBE (Fig 5B).

As preliminary investigations, the active site in pHBH was investigated using MD simulations starting from the crystal structure of the pHBH-FAD complex from 1PBE with the substrate removed. The arrangement of the identified key residues selected above could be recurrently found through the 50 ns MD, using the receptor-based scoring-function. When re-docking 4-HB in the pHBH-FAD *substrate free* complex,[66] the calculations reproduced the crystal pose of 4-HB in 1PBE. Translated to Coq6p, these calculations imply that enzyme conformations that are able to bind the substrate directly can be found by exploring *substrate free* conformational space through MD.

### *In Silico* Identification of a Substrate Access Channel in Coq6p

**Fig 6:**
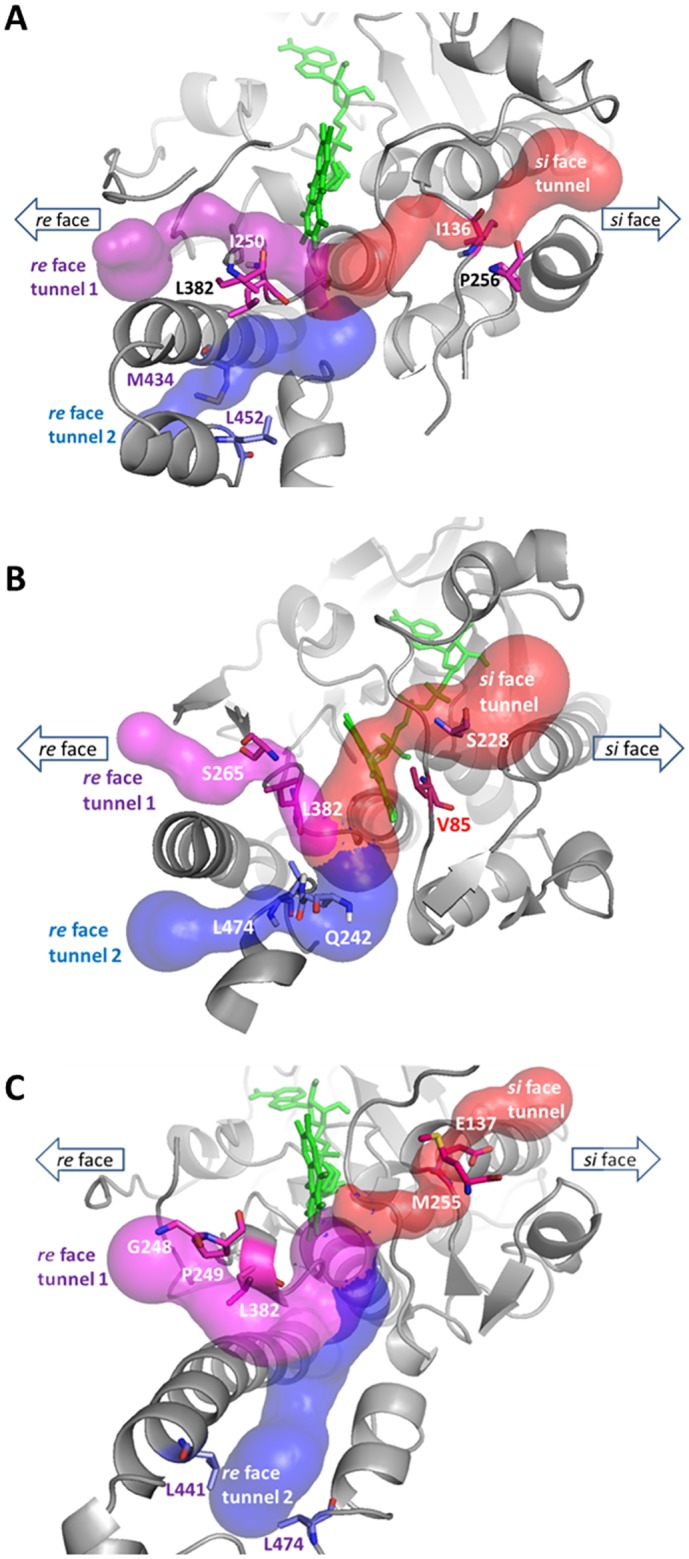
Volume rendering of the channels system identified in the resting Coq6p enzyme models: (A) Coq6p_I-TASSER model, (B) Coq6p_ROBETTA model and (C) Coq6p_MODELLER model. Three channels are identified that converge on the catalytic site close to the FAD isoalloxazine (in green stick). Two of these tunnels (1 and 2, in purple and blue, respectively) exit the enzyme from the *re* face, the other (3, in red) from the *si* face of the isoalloxazine ring. Bottleneck residues used in MD channel diameter tracking are represented as sticks.

The three Coq6p models were then analyzed for the presence of cavities for substrate binding and access to the catalytic site by computation of accessible void regions. Three distinct tunnels connecting the Coq6p surface to the putative catalytic site were identified on the pre-MD homology models (Fig 6). Two of these tunnels (1 and 2, in purple and blue, respectively) exit the enzyme from the *re* face, the other (3, in red) from the *si* face of the isoalloxazine ring.

Assuming that substrate binding does not involve significant induced fit effects, it should traverse at least one of the three channels. For each of these, a pair of residues corresponding to the bottlenecks was identified, and the corresponding distance monitored through the MD simulations (S12 Fig). MD snapshots were extracted according to the maximal values of this bottleneck distance and served as targets for automated substrate docking, using 3-hexaprenyl-4-hydroxyphenol (4-HP6) as a Q_6_ model substrate.

The choice of the model substrate as 4-HP6 was motivated as follows. In earlier studies, 3-hexaprenyl-4-hydroxybenzoate (4- HB6) has been proposed to be the substrate of Coq6p on the basis that the decarboxylation reaction of the eukaryotic Q biosynthesis pathway could occur after the C5-hydroxylation step catalyzed by Coq6p.[67] However, we have recently reported that cells lacking an active Coq6p enzyme accumulate 3-hexaprenyl-4-hydroxyphenol (4-HP6),[17] showing that the C1-decarboxylation and C1 hydroxylation reactions can occur prior to the C5-hydroxylation catalyzed by Coq6p (S13 Fig). Yet clear experimental evidence are missing to strongly support either 4-HB6 or 4-HP6 as substrate of Coq6p since no *in vitro* assay is available and the decarboxylase enzyme is not identified.[13] Moreover, in pHBH,[40] the carboxyl group of the substrate 4-hydroxybenzoate is hydrogen bonded with R214 in the active site, but Coq6p lacks such a residue (5B Fig), suggesting that a benzoate group may not be present on the aromatic head of the Coq6p substrate. Overall, these features prompted us towards 4-HP6 as a model substrate rather than 4-HB6, at variance with the known substrate of PHBH. Still, docking studies were performed also with 4-HB6 as a model substrate. While changing functional groups of the aromatic head from a hydroxyl to a carboxyl did not change the further conclusions in this study, the ensemble of 4-HB6 substrate docked poses is more diverse (S14 Fig) while in both cases the same most stable binding mode is found. This difference in calculation convergence is most probably related to the lack of recurrent stabilizing interactions between the carboxyl group of 4-HB6 and the residues of the Coq6p active site, which we assign to the absence of a PHBH R214 homolog that would facilitate the recognition of a carboxylate substrate. We therefore present here our investigation of substrate docking with 4-HP6, while our conclusions regarding the substrate access channel are similar with both models.

**Fig 7:**
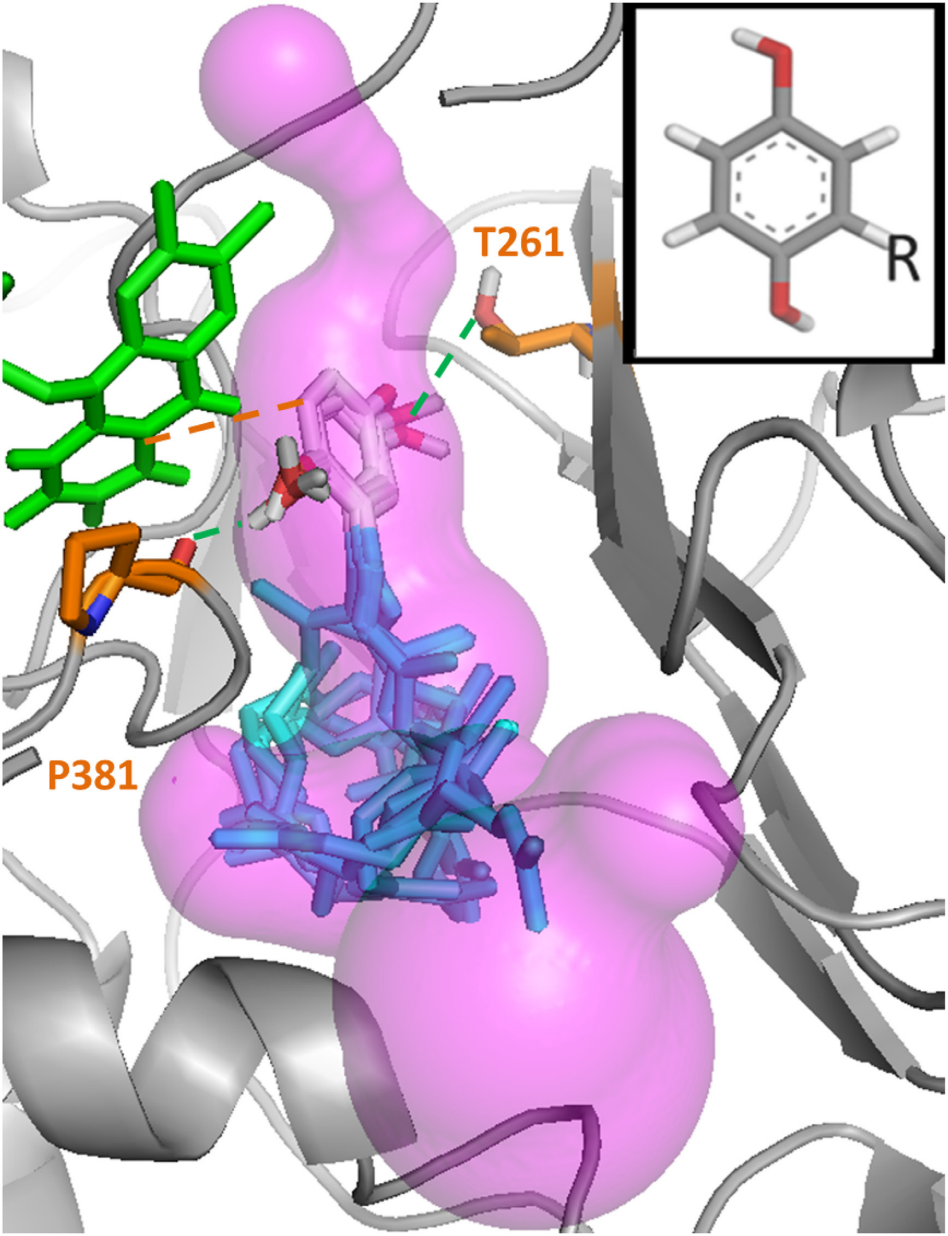
Recurrent catalytically plausible poses of the 3-hexaprenyl-4-hydroxyphenol model substrate in the *re* face tunnel 1 of the Coq6p_MODELLER model. The enzyme conformation for the substrate docking calculations was selected with the active site geometric descriptor scoring-function derived from 1PBE[40] (see Methods). The FAD co-factor is represented in green stick and the substrate in grey (aromatic head) and blue (isoprenyl chain). Several substrate poses are superimposed within the accessible volume (transparent pink) delimited by the *re* face substrate access tunnel 1. The substrate’s C1 and C6 hydroxyl groups can form hydrogen bonds (green dashed lines) with the backbone oxygen of P381 and the side chain oxygen of T261. One pose is also illustrated that shows a distance between the substrate’s C5 carbon (the hydroxylation target) and the FAD’s C4X (which bears the reactive peroxo group) of 4.7Å (dashed orange line), similar to the homologous distance observed in the 1PBE structure of 4.3Å.

It was then found that only one specific tunnel in one model (the *re* face tunnel 1 in the Coq6p_MODELLER model, Fig 6C, purple volume) produced plausible binding modes of 4-HP6, i.e. with the substrate aromatic head placed in the cavity of the putative catalytic site (Fig 7). Using the receptor-based scoring function, MD conformations from this model were further selected so as to resemble the 1PBE enzyme substrate-bound conformation as much as possible (see Methods). In a second round of calculations, the substrate was docked into these Coq6p conformations. This resulted in a systematic convergence to a single binding mode with the aromatic head close to the FAD cofactor and the hydroxyls oriented as to bind the two following residues (Fig 7): the H-bond donor, T261, and the H-bond acceptor, P381, a highly conserved residue among Coq6 enzymes (S5 Fig). The distance between the substrate C5 atom and FAD C4X atom is consistently is found at 4.7 Å (Fig 7), very similar to the corresponding 4.32 Å FAD-4-HB distance in the pHBH-FAD-4-HB complex from 1PBE.[40] The isoprenyl chain can adopt a variety of conformations traversing the channel and reaching the surface, and exhibits a number of contacts with hybrophobic residues such as P249, L253, L382, L438 and F439. Overall, these sequential series of calculations allowed us to identify the *re* face tunnel 1 as the putative substrate access channel in Coq6p.

**Fig 8:**
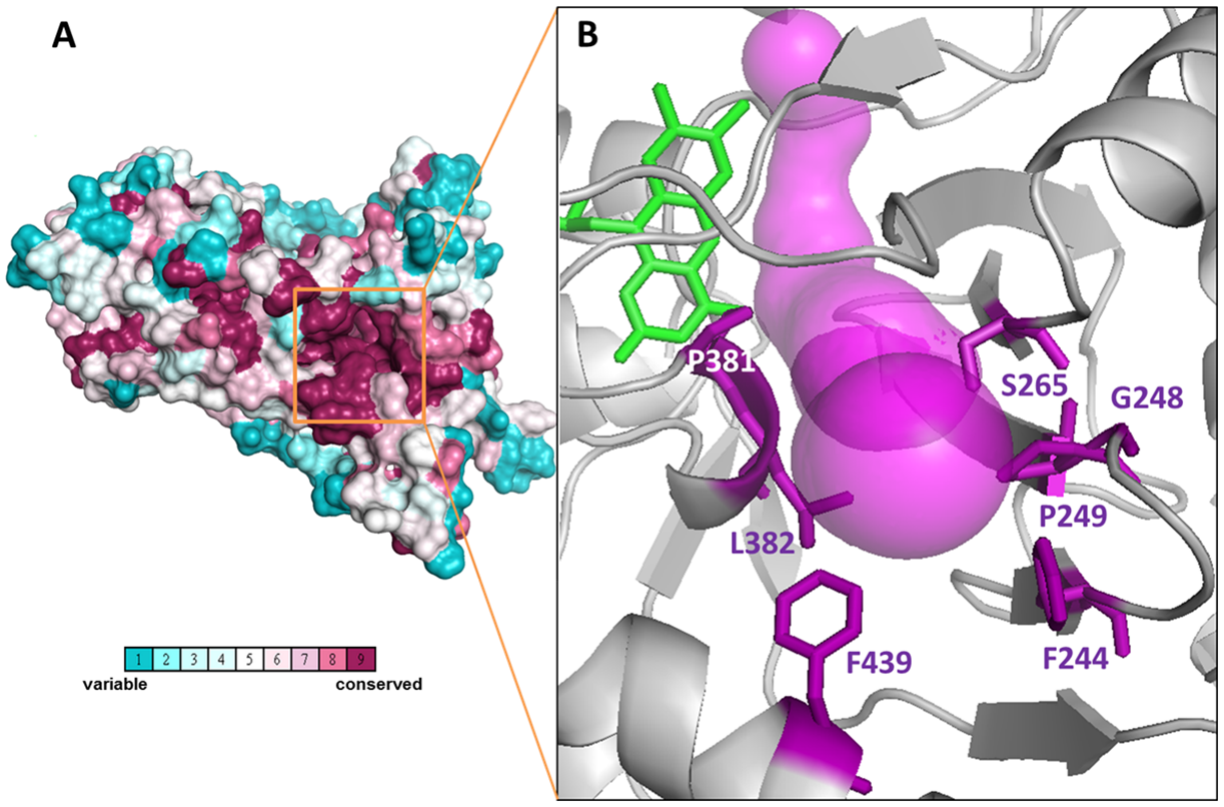
A) Evolutionary residue conservation in Coq6p as calculated by the ConSurf method [30,31] and projected onto the manual Coq6p homology model, space-filling rendering. The conservation scores can identify the FAD binding site (not visible in this view). B) Residue conservation scores also identify a group of conserved residues along the *re* face tunnel 1 (represented in magenta) reaching the surface and forming a depression (delineated by a black circle in panel A). This surface depression leads to a void volume contiguous with the catalytic site, and is lined with highly conserved residues represented in magenta sticks. For clarity, only the residues present at the tunnel entrance are shown. This set of residues is generally hydrophobic, with rare exception, such as S265. The polar residues may participate in contacting the substrate’s aromatic head as it traverses the channel to the catalytic site. FAD is represented in green stick.

Evolutionary conservation of residues lining the tunnel system of Coq6p was determined by submitting the Coq6p sequence to the ConSurf server and mapping the residue conservation scores onto the Coq6p homology model (Fig 8A). Interestingly, it identified the *re* face tunnel 1 leading to the active site as the most conserved tunnel. Furthermore, the walls of tunnel 1 are lined almost exclusively with hydrophobic and uncharged side chains, F244, P249, S265, P381, L382 and F439, (Fig 8B), which is appropriate for the channeling of the isoprenyl chain. For comparison, the 4N9X experimental structure (an UbiI homologue) was also submitted to establish a map of residue conservation, which pointed to a similarly conserved *re* face tunnel. All together, these results suggest that the hydrophobic character of this tunnel is conserved.

### *In Silico* Mutagenesis

**Fig 9:**
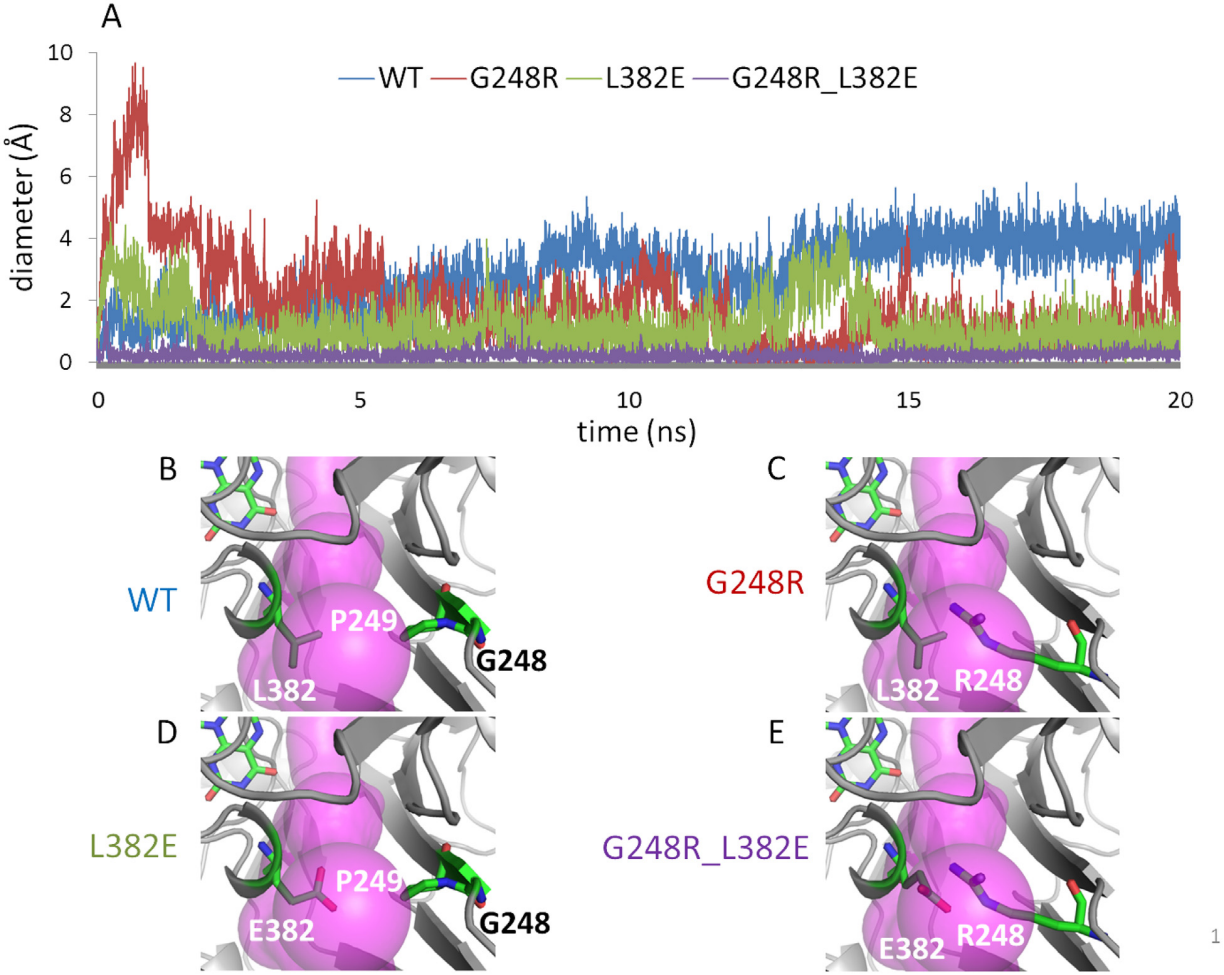
Molecular dynamics of Coq6p homology models. A): Time-evolution of substrate access channel diameter at choke points over MD runs for Coq6p_MODELLER homology model: wild-type (blue trace), Coq6p-G248R (red trace) and Coq6p-L382E (green trace) single mutants, and the Coq6p-G248R-L382E double mutant (purple trace). B): In the wild-type, the channel diameter is large and is constitutively open. C-D): The single mutants display consistently reduced channel diameters which show thermal fluctuations around open and closed states. E): The double mutant displays a channel diameter that is always below the minimum required for substrate binding. In this figure, *re* face tunnel 1 (purple volume) is oriented directly to the viewer. The pair of residues tracked of the MD calculations as bottlenecks of the channel are represented in sticks.

Several deleterious mutations in human Coq6, hCoq6, were recently reported, including R162X, G255R, A353D and W447X,[20] D208H[68] and Y412C.[59] Aligning the hCoq6 sequence with our manually curated alignment, corresponding Coq6p residues were identified and mapped onto the homology model (S15 Fig). Interestingly, the hCoq6 G255R mutation translates to a G248R mutation in Coq6p, and is positioned at the entrance of the putative *re* face substrate access tunnel 1 identified above, with the arginine sidechain pointing towards the channel lumen (Fig 9C). MD trajectories of the Coq6p-G248R mutant showed a significant reduction of the channel diameter at the choke point (Fig 9A, red line) with respect to the wild type (Fig 9A, blue line). The R248 sidechain samples blocking and non-blocking conformations, consistent with the reported reduced activity of the corresponding human mutated enzyme.[20,59] We also identified L382 (Fig 9B), a highly conserved residue which faces G248 across the channel, as a key site to introduce another mutation to block this channel. The L382 sidechain is oriented towards the lumen of the access channel and could be mutated to a larger sidechain. Its solvent exposure also suggests that mutation to a polar or charged residue may be possible. We reasoned that a mutation to glutamate, L382E, would be tolerated by the structure while allowing the formation of a salt bridge with the G248R sidechain (Fig 9E). MD calculations of the single Coq6p-L382E mutant (Fig 9A, green line, and Fig 9D) showed a significant decrease of the channel diameter when compared to the wild type. However, traversable conformations were occasionally observed during MD runs, allowing us to dock the model substrate. Turning to the Coq6p-G248R-L382E double mutant, MD calculations predicted the formation of a stable salt bridge spanning the channel lumen and causing a complete and persistent blocking of the channel over the 20 ns trajectory (Fig 9A, purple line). Overall, these results show that single mutations partially block the channel, whereas the double mutation causes a complete blockage.

### *In Vivo* Mutagenesis Results

**Fig 10:**
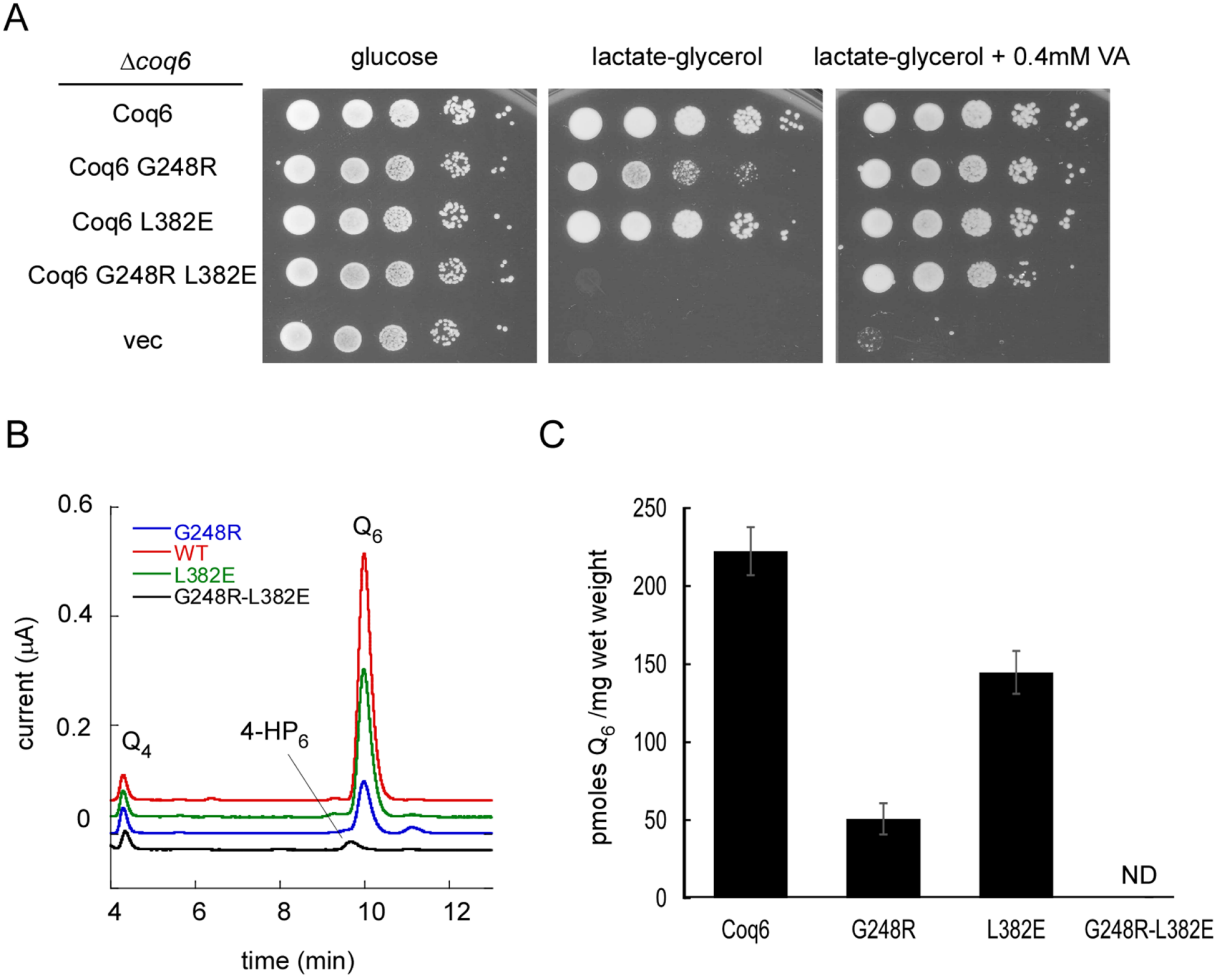
A) 10 fold serial dilution of the Δcoq6 strain carrying an empty plasmid (vec) or plasmids coding for Coq6p, Coq6p-G248R, Coq6p-L382E and Coq6p-G248R-L382E. The plates contained YNB-pABA agar medium supplemented with the indicated carbon source and vanillic acid (VA) or not. The plates were imaged after incubation at 30°C for 2 days (glucose) or 6 days (lactate-glycerol). B) Representative electrochromatogram of lipid extracts from Δcoq6 cells expressing either Coq6p, Coq6p-G248R, Coq6p-L382E and Coq6p-G248R-L382E (1 mg of cells). The elution position of the Q_4_ standard, of 3-hexaprenyl-4-hydroxyphenol (4-HP6) and Q_6_ are indicated. C) Q6 amounts (in pmoles per mg of wet weight) in Δcoq6 cells expressing either Coq6p, Coq6p-G248R, Coq6p-L382E or Coq6p-G248R-L382E. Cells were grown in YNB–pABA 2% lactate-glycerol containing 10 μM 4HB. The results are the average of 3–4 independent experiments and error bars represent standard deviation. ND, not detected.

To corroborate our *in silico* predictions, site-directed mutagenesis was performed to test the role of the G248 and L382 residues by studying the single mutants G248R and L382E, as well as the double mutant G248R-L382E. The effect of these different mutations on Coq6p activity was tested *in vivo*. Contrary to growth on the fermentable glucose medium, growth on the respiratory medium containing lactate-glycerol (LG) requires a functional respiratory chain and is thus dependent on ubiquinone. Accordingly, the Δcoq6 strain is unable to grow on LG medium unless it is complemented with a plasmid expressing Coq6p (Fig 10A). The growth provided by Coq6p-L382E was comparable to that of Coq6p, but Coq6p-G248R was slightly compromised (Fig 10A). The Δcoq6 strain expressing Coq6p-G248R-L382E was unable to grow on LG medium showing that the L382E and G248R mutations have an additive effect. Addition of vanillic acid (VA) to the growth medium is able to restore Q_6_ biosynthesis in Coq6-deficient cells by bypassing the deficient C5-hydroxylation reaction, provided that the Q biosynthetic complex is stable which requires a stable Coq6p polypeptide.[17] Non-empty vectors expressing any of the three Coq6p mutant proteins allowed growth on LG medium + VA (Fig 10A), supporting that the Coq6p-G248R-L382E is stable. HPLC-ECD analysis of the Q content correlated with the growth phenotypes and showed that Q was undetectable in cells expressing Coq6p-G248R-L382E (Fig 10B). These cells accumulated 4-HP6 which is characteristic of a C5-hydroxylation defect but forms only when the CoQ synthome is assembled, supporting again that Coq6p-G248R-L382E accumulates *in vivo* as a stable polypeptide. The Q levels measured in cells expressing Coq6p-G248R and Coq6p-L382E were intermediate (Fig 10B and 10C). Altogether, these results show that the G248R and L382E mutations decrease Coq6p activity to some extent while the combination of both mutations completely inactivates the protein without affecting its stability. These data are consistent with the theoretical prediction of the substrate channel being blocked by the proposed interaction between R248 and E382 residues.

## Discussion

Biochemical results provide evidence that Coq6p is a flavoprotein, using FAD as a co-factor. A sequential computational strategy was then adopted comprising the construction of homology models of Coq6p, followed by MD calculations of the FAD-Coq6p complexes. We used these models to get insight into the catalytic site structure and a tunnel system in Coq6p. A specific tunnel was identified and experimentally substantiated through *in vivo* studies of selected mutants.

One important limitation of homology modeling is the availability of templates with sufficiently high sequence identities. While operating in a regime of low sequence identity, the sequence-based searched templates used for Coq6p homology models (i.e. 4N9X, 4K22,[24] 2X3N,[27] and 1PBE,[40]) share the same Rossmann-fold of monooxygenase structures, belonging to the class A of flavoproteins. [69,70]Importantly, all three FAD-Coq6p homology models display well-formed α-helix loops as part of the highly conserved GDAxH motif (highlighted in orange in S4 Fig), directly inherited from their respective template in that region. Through MD calculations, FAD in complex with Coq6p recapitulates a number of canonical enzyme-cofactor contacts (Fig 4). When compared to 2X3N,[27] a FAD-cocrystallized structure, the ribityl chain is H-bonded to the side-chain of D374, (D292 in 2X3N) and the ribose is H-bonded to the side-chain of D61, (Q35 in 2X3N). Also, the negatively charged pyrophosphate group is electrostatically complemented by the α1 helix dipole of the *β/α/β* fold at the N-terminus as in 2X3N.

Turning to structure-function relationships, studies of pHBH’s catalytic cycle indicate that the substrate enters the active site through a channel, the active site being shielded from solvent.[71] We reasoned that the physical pathway of the aromatic head entry and isoprenyl tail binding is likely to be also a tunnel traversing the Coq6p protein. Calculations on the resting Coq6p models (prior MD) show that they share a general multiple channel system, with three distinct tunnels converging to the highly conserved active site (Fig 6), a buried volume immediately facing FAD.

The traversability of Coq6p channel system by the substrate was assessed computationally. Here, we postulate a conformational pre-selection mechanism (as opposed to induced-fit) whereby conformations of Coq6p compatible with substrate binding might be accessible through MD calculations on the substrate-free enzyme.[72] The key outcome of these calculations is that *re* face tunnel 1 may be the Coq6p access channel for its substrate (Figs 7–9). Interestingly, a recently reported crystal structure of a PHBH-family flavoprotein, namely the 3-Hydroxybenzoate 6-Hydroxylase (4BJY),[73] features a bound lipid positioned in the *re* face channel 2 identified in all Coq6p models. The comparison of both enzymes (S16 Fig) highlights how the orientation of the helix 12 determines the access of the lipidic chain either through the C-terminus “below” helix 12 (4BJY) or through the channel “above” helix 12 (Coq6p). Turning to Coq6p, the *re* face tunnel 2 was discarded since no plausible poses for 4HP6 was found in the present work. Still, the aromatic head of 4-HP6 docked in Coq6p (tunnel 1) occupies the same region than the substrate of 4BJY (as inferred from 4BK1, a mutant co-crystallized with its substrate and lipid), i.e. in a catalytically plausible pose in the vicinity of the FAD isoalloxazine ring, highlighting the similar function of these two proteins belonging to the same family. Importantly, the *re* face tunnel 1 turned out to be the most conserved one and furthermore it is lined with hydrophobic residues appropriate for contact with the isoprenyl tail of the substrate.

This drove us to perform *in silico* mutagenesis at its bottleneck and propose single and double-mutations to potentially hinder substrate binding. The experimental *in vivo* findings, i.e. the reduced Q6 biosynthesis of the single and double mutants, provide *a posteriori* validation of our *in silico* mutagenesis, and overall of the computational strategy including our conformational pre-selection hypothesis.

This work provides the first detailed structural information of an important and highly conserved enzyme of ubiquinone biosynthesis in the absence of crystallographic data. First, it demonstrates that Coq6p is a FAD enzyme and thus belongs to the FAD-dependent mono-oxygenase family. Second, in order to accommodate a bulky hydrophobic substrate, it has evolved a channel and a cavity to specifically direct the substrate towards the catalytic site. Future work should address the catalytic activity of Coq6p since so far we and others failed to find the proper conditions for detecting *in vitro* enzyme activity. This is not surprising considering the difficulty related to the synthesis of the substrate (highly hydrophobic), to the complexity of the redox system associated to Coq6p activity [16] and the likely requirement for protein partners within CoQ synthome.[12] The availability of a structural model for Coq6p makes it possible to consider further computational approaches regarding its integration in more complex interactions with other proteins and with the membrane. As exemplified here, *in silico* mutagenesis studies can be coupled to *in vivo* confirmation to explore functional hypothesis.

## Supporting Information

S1 Text Comments on the GDAxH loop compatible with FAD binding. (DOCX)

S1 Table Oligonucleotides used in the present study. (DOCX)

S2 Table RMSDs (Å) of Coq6p homology models prior MD simulations and related templates (calculated with Superpose version 1.0, http://wishart.biology.ualberta.ca/SuperPose/help.html). (DOCX)

S1 Fig Optimized DNA sequence of the coq6 gene from Saccharomyces cerevisiae and its translation in amino-acids. The *malE* protein from *E*.*coli* is in blue; the *coq6* protein is in red.
(DOCX)

S2 Fig Determination of the flavin cofactor of Coq6p-MBP. HPLC chromatograms (A_265nm_) from a solution of 30 μM Coq6p-MBP after denaturation (100°C, 10 min) (green curve) and FAD and FMN standards (20 μM) (blue and red curves respectively). HPLC was carried out on a Hypersil Gold C-18 analytical column (1.9 μm, 2.1 x 50 mm).
(DOCX)

S3 Fig Sequence alignment of Coq6p with 2X3N, 4N9X, 4K22 and 1PBE complete sequences. Sequence alignment of Coq6p with the monooxygenase in *Erwinia carotovora* (PDB entry 4N9X), UbiI in *E*. *coli* (PDB entry 4K22), PqsI in *Pseudomonas aeruginosa* (PDB entry 2X3N) and pHBH in *Pseudomonas fluorescens* (PDB entry 1PBE) reveals low sequence identities to Coq6p: 28.32%, 27.30%, 20.30% and 18.51% respectively. Alignment was made with ClustalW with the ClustalX colour scheme. For further details, see: http://ekhidna.biocenter.helsinki.fi/pfam2/clustal_colours.
(DOCX)

S4 Fig Structural comparison of the structural templates used in this work, 2X3N, 4K22, 4N9X, and 1PBE. All four templates exhibit similar overall tertiary structures with a typical Rossmann-like β/α/β fold in addition to an FAD-binding domain. The C-terminus, which is missing in the 4K22 and 2X3N templates, exhibits an extended structure in 4N9X or a more compact one in 1PBE. It is noteworthy that the FAD co-factor is co-crystallized in 2X3N and 1PBE, making the latter valuable templates for a model of the FAD-dependent Coq6p enzyme. Main Chain RMSD WRT 1PBE: 2X3N (3.27 Å), 4N9X (5.45 Å), 4K22 (6.84 Å). Alpha carbon RMSD WRT 1PBE: 2X3N (3.32 Å), 4N9X (5.49 Å), 4K22 (6.83 Å).
(DOCX)

S5 Fig Alignment as extracted from the Multiple Sequence Alignment of Coq6p with 119 homologues as calculated by ConSurf. Alignment was made with ConSurf and colored with the ClustalX colour scheme. The Coq6p insert consists of 51 residues (as identified in Fig 2) and is highlighted in the purple frame. The sequence of human Coq6 was manually added to the reference MSA.
(DOCX)

S6 Fig Alignments used for the generation of the Coq6p homology models. A) Manually curated alignment used for the construction of Coq6p_MODELLER model. B) ROBETTA template construction alignment. C) Alignments used for the generation of the Coq6p homology models. I-TASSER template construction alignment.
(DOCX)

S7 Fig Time evolution over MD calculations of regional RMSDs for selected structural elements of Coq6p models. Red: Coq6p_ITASSER model; Green: Coq6p_ROBETTA model; Blue: Coq6p_MODELLER model.
(DOCX)

S8 Fig Secondary structure timeline analysis (50 ns) as computed by the timeline plugin contained in VMD for the Coq6p_I-TASSER model. In the graphic, the β-sheet components turn (T) and extended conformation (E) are represented in teal and yellow respectively; isolated bridges are in dark yellow; degrees of helix are in pink (α-helix), blue (3–10 helix) and red (π-helix); random coils are in white.
(DOCX)

S9 Fig Secondary structure timeline analysis (50 ns) as computed by the timeline plugin contained in VMD for the Coq6p_MODELLER model. In the graphic, the β-sheet components turn (T) and extended conformation (E) are represented in teal and yellow respectively; isolated bridges are in dark yellow; degrees of helix are in pink (α-helix), blue (3–10 helix) and red (π-helix); random coils are in white.
(DOCX)

S10 Fig Secondary structure timeline analysis (50 ns) as computed by the timeline plugin contained in VMD for the Coq6p_ROBETTA model. In the graphic, the β-sheet components turn (T) and extended conformation (E) are represented in teal and yellow respectively; isolated bridges are in dark yellow; degrees of helix are in pink (α-helix), blue (3–10 helix) and red (π-helix); random coils are in white.
(DOCX)

S11 Fig Comparison of conformations of the three Coq6p models before and after 20 ns MD. Colors refer to helix 8 (yellow) and sub-sections of the Coq6p insert (N-terminal side of the insert in cyan, ascending part in red, C-terminal side of the insert in purple) as annotated in the alignment with 2X3N shown below. FAD is not shown for clarity.
(DOCX)

S12 Fig MD time-evolution in nanoseconds of the selected interatomic distances at choke points of tunnels 1, 2 and 3 in the three Coq6p models. Top: tunnel 1; middle: tunnel 2; bottom: tunnel 3. Left: Coq6p_I-TASSER model; Middle: Coq6p_ROBETTA model; left: Coq6p_MODELLER model.
(DOCX)

S13 Fig S. cerevisiae Q6 biosynthetic pathway. 4-hydroxybenzoic acid (4-HB) is prenylated by Coq2 to form 3-hexaprenyl-4-hydroxybenzoic acid (4-HB_6_). R represents the hexaprenyl tail on the biosynthetic products downstream of 4-HB_6_ and the numbering of the aromatic carbon atoms used in this study is shown on the reduced form of Q_6_, Q_6_H_2_. Upon inactivation of coq6 (path in red), 4-HB_6_ is decarboxylated (dashed arrow) and hydroxylated at position C1, yielding 3-hexaprenyl-4-hydroxyphenol (4-HP_6_). Vanillic acid (VA, in green) contains a methoxyl group on C5 and thus bypasses the C5-hydroxylation defect in coq6-deficient strains because it is converted into Q_6_ after its prenylation by Coq2 (see Ozeir et al. (2011) *Chem Biol*. 18, 1134–1142).
(DOCX)

S14 Fig Docked poses of 3-hexaprenyl-4-hydroxyphenol model substrate (4-HB6) in the Coq6p_MODELLER model. The enzyme conformation for the substrate docking calculations was selected with the active site geometric descriptor scoring-function derived from 1PBE (see Methods). The FAD co-factor is represented in green stick and the substrate in grey (aromatic head) and blue (isoprenyl chain). Several substrate poses are superimposed within the accessible volume (transparent pink) delimited by the *re* face substrate access tunnel 1.
(DOCX)

S15 Fig Schematic ribon representation of Coq6p with the positions of mutated residues reported in literature for hCoq6. The hCoq6-Coq6p alignement based on the multiple sequence alignement of 119 Coq6p homologues was used for the identification of homologous residues, as follows: the mutations reported by Heeringa et al. (2011), R162X, G255R and A353D are identified as located at residues K155, G248, A361 in Coq6p. The homologous residue for D208H mutation reported for hCoq6 by Zhang et al. (2014) is D201 in Coq6p. The Y412C mutation in hCoq6 reported by Doimo et al. (2014) translates into Coq6p F420.
(DOCX)

S16 Fig Comparison of the positioning of 4HP6 model substrate in Coq6p (A) and that of the bound lipid—a mixture of phosphatidylglycerol and phosphatidylethanol-amine- in 4BJY (B). The FAD co-factor is represented in green sticks and the lipid/4HP6 in sticks within blue transparent volumes. In 4BJY the bound lipid enters through the large triangle formed by the C-terminus, with the lipid passing below Helix 12 (named *re* face channel 2 in the present work). In Coq6p, docking calculations and residue conservation suggest passage above Helix 12 (named *re* face channel 1 in the present work). The dashed circle in A highlights the position of the aromatic head of 4HP6. The dashed circle in B highlights the position of 3-Hydroxybenzoate as obtained in the co-crystallized 4BK1 crystal structure.
(DOCX)
