# Supplementary material for: Coenzyme Q Biosynthesis: Evidence for a Substrate Access Channel in the FAD-Dependent Monooxygenase Coq6
Source: PLoS Comput Biol. 2016 Jan 25;12(1):e1004690. doi: 10.1371/journal.pcbi.1004690 (PMC4726752; doi:10.1371/journal.pcbi.1004690)
Supplement: S1 Text — (DOCX) [file pcbi.1004690.s001.docx]

**S1 Text: Comments on the GDAxH loop compatible with FAD binding.** A comparative inspection of the templates (and all other Rossmann-fold monooxygenase structures from the PDB) reveals that whenever this class of enzyme is co-crystallized with FAD, the FAD binding-loop conforms to a single turn of α-helix. There are no structures where this loop is non-helical and bound to FAD, suggesting that a catalytically competent Coq6 may have its FAD binding-loop in an α-helix as well. Among the template set, 4K22 and 4N9X, which were not co-crystallized with FAD, display distorted FAD binding loops, whereas 2X3N and 1PBE display well-formed alpha helix loops and were crystallized with FAD. In addition, DALI search results indicate a closer structural match between 4K22 and 2X3N than between 4K22 and 1PBE. We inferred that since yCoq6 is a functional homolog of 4K22, it is likely to also be a closer structural match to 2X3N. This led us to select 2X3N as a template for the N-terminal majority of yCoq6 (up to residue 342). The C-terminus is a region of variable conformation among the template set, and misses coordinates both 4K22 and 2X3N structures, due to proteolytic truncation and high mobility, respectively. The C-terminus is important because it is proximal to the active site and is likely to be involved in substrate binding according to ConSurf analysis, making 4N9X the best template for this region as being crystallized in its full-length form.
