# Supplementary material for: Coenzyme Q Biosynthesis: Evidence for a Substrate Access Channel in the FAD-Dependent Monooxygenase Coq6
Source: PLoS Comput Biol. 2016 Jan 25;12(1):e1004690. doi: 10.1371/journal.pcbi.1004690 (PMC4726752; doi:10.1371/journal.pcbi.1004690)
Supplement: S1 Table — (DOCX) [file pcbi.1004690.s002.docx]

**S1 Table. Oligonucleotides used in the present study.**

| 3′L382Ecoq6 | 5’- CCATGTTTAACCCCTGTCCAGCTTCAGGATGTGTCG-3’ |
| --- | --- |
| 5′L382Ecoq6 | 5’- GCACATACGACACATCCTGAAGCTGGACAGGGG-3’ |
| SacI coq6 | 5’- GCCGTCTAAGTGGAGCTCAGGAGGC-3’ |
| HindIII coq6 | 5’- CCATGATTACGCCAAGCTTGGCCGC-3’ |
