# Supplementary material for: Coenzyme Q Biosynthesis: Evidence for a Substrate Access Channel in the FAD-Dependent Monooxygenase Coq6
Source: PLoS Comput Biol. 2016 Jan 25;12(1):e1004690. doi: 10.1371/journal.pcbi.1004690 (PMC4726752; doi:10.1371/journal.pcbi.1004690)
Supplement: S2 Table — (DOCX) [file pcbi.1004690.s003.docx]

**S2 Table. RMSDs (Å) of Coq6p homology models prior MD simulations and related templates (calculated with Superpose version 1.0, http://wishart.biology.ualberta.ca/SuperPose/help.html).**

| **Reference structure** | **Comparison structure** | **RMSD Ca** | **RMSD Main Chain** |
| --- | --- | --- | --- |
| Coq6_MODELLER | Coq6_ITASSER | 6,39 | 6,33 |
| Coq6_MODELLER | Coq6_ROBETTA | 6,53 | 6,53 |
| Coq6_ITASSER | Coq6_ROBETTA | 7,61 | 7,55 |
| Coq6_MODELLER | 1PBE | 15,47 | 15,36 |
| 1PBE | 2X3N | 13,18 | 13,14 |
| 1PBE | 4K22 | 7,73 | 7,66 |
| 1PBE | 4N9X | 7,42 | 7,34 |
