# Supplementary material for: Coenzyme Q Biosynthesis: Evidence for a Substrate Access Channel in the FAD-Dependent Monooxygenase Coq6
Source: PLoS Comput Biol. 2016 Jan 25;12(1):e1004690. doi: 10.1371/journal.pcbi.1004690 (PMC4726752; doi:10.1371/journal.pcbi.1004690)
Supplement: S1 Fig — The malE protein from E.coli is in blue; the coq6 protein is in red. (DOCX) [file pcbi.1004690.s004.docx]

5’ATGAAAATCGAAGAAGGTAAACTGGTAATCTGGATTAACGGCGATAAAGGCTATAACGGTCTCGCTGAAGTCGGTAAGAAATTCGAGAAAGATACCGGAATTAAAGTCACCGTTGAGCATCCGGATAAACTGGAAGAGAAATTCCCACAGGTTGCGGCAACTGGCGATGGCCCTGACATTATCTTCTGGGCACACGACCGCTTTGGTGGCTACGCTCAATCTGGCCTGTTGGCTGAAATCACCCCGGACAAAGCGTTCCAGGACAAGCTGTATCCGTTTACCTGGGATGCCGTACGTTACAACGGCAAGCTGATTGCTTACCCGATCGCTGTTGAAGCGTTATCGCTGATTTATAACAAAGATCTGCTGCCGAACCCGCCAAAAACCTGGGAAGAGATCCCGGCGCTGGATAAAGAACTGAAAGCGAAAGGTAAGAGCGCGCTGATGTTCAACCTGCAAGAACCGTACTTCACCTGGCCGCTGATTGCTGCTGACGGGGGTTATGCGTTCAAGTATGAAAACGGCAAGTACGACATTAAAGACGTGGGCGTGGATAACGCTGGCGCGAAAGCGGGTCTGACCTTCCTGGTTGACCTGATTAAAAACAAACACATGAATGCAGACACCGATTACTCCATCGCAGAAGCTGCCTTTAATAAAGGCGAAACAGCGATGACCATCAACGGCCCGTGGGCATGGTCCAACATCGACACCAGCAAAGTGAATTATGGTGTAACGGTACTGCCGACCTTCAAGGGTCAACCATCCAAACCGTTCGTTGGCGTGCTGAGCGCAGGTATTAACGCCGCCAGTCCGAACAAAGAGCTGGCAAAAGAGTTCCTCGAAAACTATCTGCTGACTGATGAAGGTCTGGAAGCGGTTAATAAAGACAAACCGCTGGGTGCCGTAGCGCTGAAGTCTTACGAGGAAGAGTTGGCGAAAGATCCACGTATTGCCGCCACTATGGAAAACGCCCAGAAAGGTGAAATCATGCCGAACATCCCGCAGATGTCCGCTTTCTGGTATGCCGTGCGTACTGCGGTGATCAACGCCGCCAGCGGTCGTCAGACTGTCGATGAAGCCCTGAAAGACGCGCAGACTAATTCGAGCTCGAACAACAACAACAATAACAATAACAACAACCTCGGGATCGAGGGAAGGATTTCAGAATTCGGATCCTTCTTTTCAAAAGTTATGCTTACTCGGCGTATTTTGGTGCGCGGTTTGGCAACAGCCAAATCTTCAGCTCCAAAGTTAACAGATGTATTAATCGTAGGTGGGGGTCCTGCAGGTTTGACTTTAGCTGCATCGATTAAGAATTCTCCGCAATTAAAAGATTTAAAGACAACTTTAGTCGATATGGTGGACTTAAAAGATAAATTATCGGACTTTTATAATTCACCGCCAGATTATTTTACGAACCGCATTGTCAGTGTTACGCCTCGCTCTATTCATTTTCTTGAGAATAACGCTGGGGCAACTTTGATGCATGACCGCATTCAAAGTTATGACGGACTCTATGTCACGGACGGTTGTTCTAAGGCTACTTTGGATCTGGCACGCGACTCCATGCTTTGTATGATTGAAATTATTAATATTCAGGCCTCCTTATACAACCGCATTTCTCAGTACGATTCAAAGAAGGACTCCATTGATATTATTGATAATACAAAAGTTGTCAATATTAAACACAGCGACCCTAATGATCCATTGTCCTGGCCTTTAGTCACTCTTTCCAATGGTGAAGTATACAAGACGCGCTTGTTAGTGGGTGCAGACGGGTTCAATTCTCCTACCCGCCGCTTTTCCCAGATCCCATCTCGCGGCTGGATGTATAATGCCTATGGTGTTGTGGCCAGCATGAAGTTAGAGTATCCTCCGTTTAAATTACGTGGCTGGCAGCGTTTCTTACCGACTGGTCCAATTGCACACTTACCGATGCCTGAAAATAATGCTACTTTAGTCTGGAGTTCATCTGAACGTTTATCGCGCCTTTTGTTGTCATTACCTCCAGAATCATTCACTGCACTTATCAATGCTGCTTTTGTCTTGGAAGACGCAGACATGAACTACTATTACCGTACATTGGAAGATGGCTCTATGGACACCGATAAATTGATTGAAGATATTAAATTCCGCACTGAGGAAATTTACGCCACGTTGAAGGACGAGTCGGATATTGATGAAATTTACCCACCACGCGTTGTTAGTATTATCGATAAGACACGCGCACGTTTTCCGTTAAAATTAACACATGCAGATCGTTATTGCACTGATCGCGTTGCCCTTGTTGGGGACGCAGCACATACGACACATCCTCTCGCTGGACAGGGGTTAAACATGGGGCAAACAGACGTTCATGGTTTAGTATACGCTTTAGAAAAGGCAATGGAACGCGGTTTAGACATTGGCTCTTCATTGAGCTTAGAACCGTTTTGGGCAGAACGTTATCCGTCGAACAACGTTTTATTAGGAATGGCGGATAAATTATTTAAATTATATCACACTAATTTTCCTCCTGTGGTAGCCTTACGCACTTTTGGTTTGAATCTGACGAATAAGATCGGTCCAGTTAAGAATATGATCATTGACACATTAGGAGGAAATGAGAAATAA 3’

MKIEEGKLVIWINGDKGYNGLAEVGKKFEKDTGIKVTVEHPDKLEEKFPQVAATGDGPDIIFWAHDRFGGYAQSGLLAEITPDKAFQDKLYPFTWDAVRYNGKLIAYPIAVEALSLIYNKDLLPNPPKTWEEIPALDKELKAKGKSALMFNLQEPYFTWPLIAADGGYAFKYENGKYDIKDVGVDNAGAKAGLTFLVDLIKNKHMNADTDYSIAEAAFNKGETAMTINGPWAWSNIDTSKVNYGVTVLPTFKGQPSKPFVGVLSAGINAASPNKELAKEFLENYLLTDEGLEAVNKDKPLGAVALKSYEEELAKDPRIAATMENAQKGEIMPNIPQMSAFWYAVRTAVINAASGRQTVDEALKDAQTNSSSNNNNNNNNNNLGIEGRISEFGSFFSKVMLTRRILVRGLATAKSSAPKLTDVLIVGGGPAGLTLAASIKNSPQLKDLKTTLVDMVDLKDKLSDFYNSPPDYFTNRIVSVTPRSIHFLENNAGATLMHDRIQSYDGLYVTDGCSKATLDLARDSMLCMIEIINIQASLYNRISQYDSKKDSIDIIDNTKVVNIKHSDPNDPLSWPLVTLSNGEVYKTRLLVGADGFNSPTRRFSQIPSRGWMYNAYGVVASMKLEYPPFKLRGWQRFLPTGPIAHLPMPENNATLVWSSSERLSRLLLSLPPESFTALINAAFVLEDADMNYYYRTLEDGSMDTDKLIEDIKFRTEEIYATLKDESDIDEIYPPRVVSIIDKTRARFPLKLTHADRYCTDRVALVGDAAHTTHPLAGQGLNMGQTDVHGLVYALEKAMERGLDIGSSLSLEPFWAERYPSNNVLLGMADKLFKLYHTNFPPVVALRTFGLNLTNKIGPVKNMIIDTLGGNEK

**S1 Fig.** **Optimized DNA sequence of the *coq6* gene from Saccharomyces cerevisiae and its translation in amino-acids**. The *malE* protein from *E.coli* is in blue; the *coq6* protein is in red.
