# Supplementary material for: Coenzyme Q Biosynthesis: Evidence for a Substrate Access Channel in the FAD-Dependent Monooxygenase Coq6
Source: PLoS Comput Biol. 2016 Jan 25;12(1):e1004690. doi: 10.1371/journal.pcbi.1004690 (PMC4726752; doi:10.1371/journal.pcbi.1004690)
Supplement: S2 Fig — HPLC chromatograms (A265nm) from a solution of 30 μM Coq6p-MBP after denaturation (100°C, 10 min) (green curve) and FAD and FMN standards (20 μM) (blue and red curves respectively). HPLC was carried out on a Hypersil Gold C-18 analytical column (1.9 μm, 2.1 x 50 mm). (DOCX) [file pcbi.1004690.s005.docx]

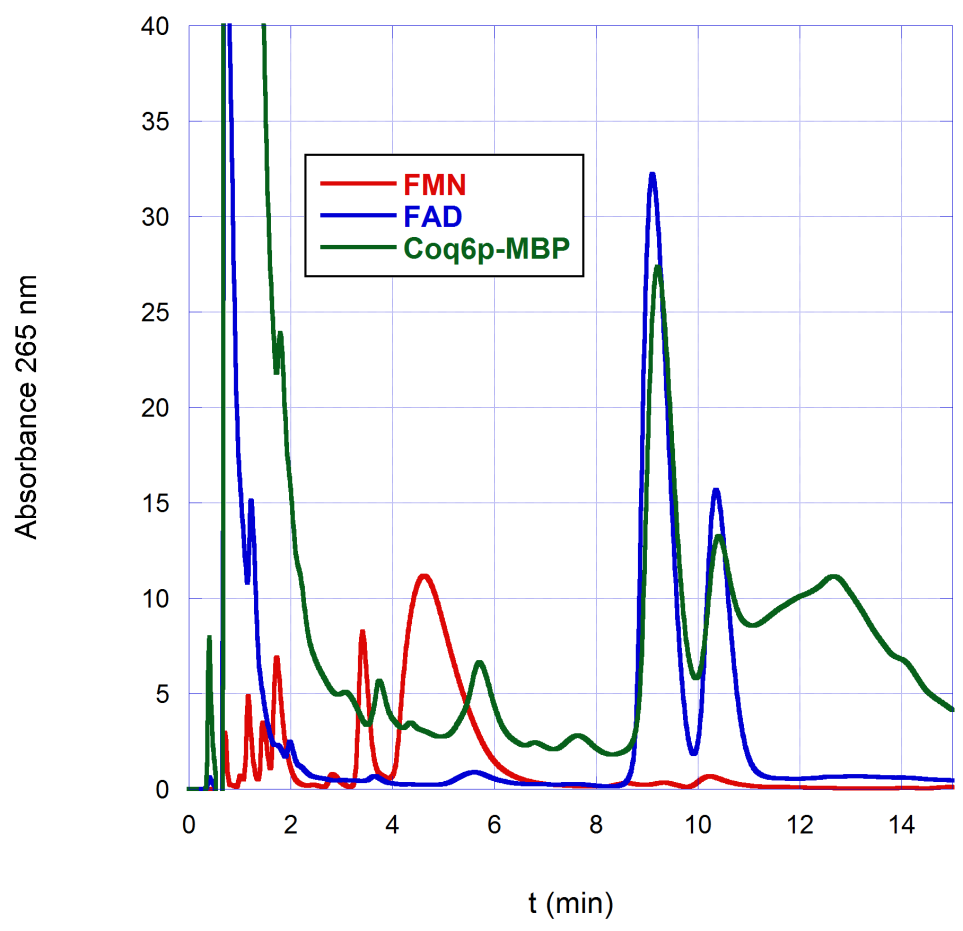


**S2 Fig. Determination of the flavin cofactor of Coq6p-MBP.** HPLC chromatograms (A_265nm_) from a solution of 30 µM Coq6p-MBP after denaturation (100°C, 10 min) (green curve) and FAD and FMN standards (20 µM) (blue and red curves respectively). HPLC was carried out on a Hypersil Gold C-18 analytical column (1.9 µm, 2.1 x 50 mm).
