# Supplementary material for: Coenzyme Q Biosynthesis: Evidence for a Substrate Access Channel in the FAD-Dependent Monooxygenase Coq6
Source: PLoS Comput Biol. 2016 Jan 25;12(1):e1004690. doi: 10.1371/journal.pcbi.1004690 (PMC4726752; doi:10.1371/journal.pcbi.1004690)
Supplement: S3 Fig — Sequence alignment of Coq6p with the monooxygenase in Erwinia carotovora (PDB entry 4N9X), UbiI in E. coli (PDB entry 4K22), PqsI in Pseudomonas aeruginosa (PDB entry 2X3N) and pHBH in Pseudomonas fluorescens (PDB entry 1PBE) reveals low sequence identities to Coq6p: 28.32%, 27.30%, 20.30% and 18.51% respectively. Alignment was made with ClustalW with the ClustalX colour scheme. For further details, see: http://ekhidna.biocenter.helsinki.fi/pfam2/clustal_colours. (DOCX) [file pcbi.1004690.s006.docx]

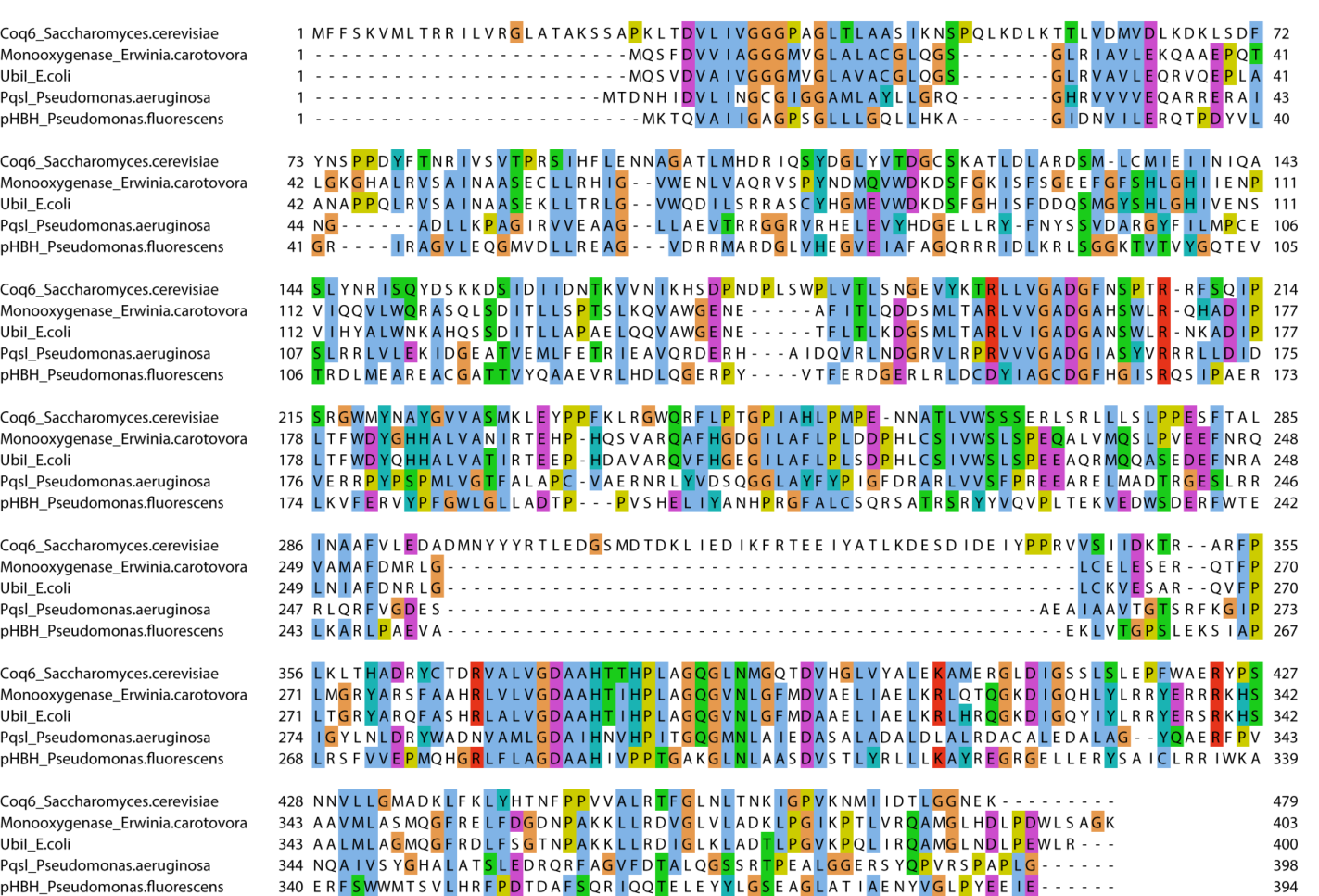


**S3 Fig. Sequence alignment of Coq6p with 2X3N, 4N9X, 4K22 and 1PBE complete sequences.** Sequence alignment of Coq6p with the monooxygenase in *Erwinia carotovora* (PDB entry 4N9X), UbiI in *E. coli* (PDB entry 4K22), PqsI in *Pseudomonas aeruginosa* (PDB entry 2X3N) and pHBH in *Pseudomonas fluorescens* (PDB entry 1PBE) reveals low sequence identities to Coq6p: 28.32%, 27.30%, 20.30% and 18.51% respectively. Alignment was made with ClustalW with the ClustalX colour scheme. For further details, see: <http://ekhidna.biocenter.helsinki.fi/pfam2/clustal_colours>.
