# Supplementary material for: Coenzyme Q Biosynthesis: Evidence for a Substrate Access Channel in the FAD-Dependent Monooxygenase Coq6
Source: PLoS Comput Biol. 2016 Jan 25;12(1):e1004690. doi: 10.1371/journal.pcbi.1004690 (PMC4726752; doi:10.1371/journal.pcbi.1004690)
Supplement: S4 Fig — All four templates exhibit similar overall tertiary structures with a typical Rossmann-like β/α/β fold in addition to an FAD-binding domain. The C-terminus, which is missing in the 4K22 and 2X3N templates, exhibits an extended structure in 4N9X or a more compact one in 1PBE. It is noteworthy that the FAD co-factor is co-crystallized in 2X3N and 1PBE, making the latter valuable templates for a model of the FAD-dependent Coq6p enzyme. Main Chain RMSD WRT 1PBE: 2X3N (3.27 Å), 4N9X (5.45 Å), 4K22 (6.84 Å). Alpha carbon RMSD WRT 1PBE: 2X3N (3.32 Å), 4N9X (5.49 Å), 4K22 (6.83 Å). (DOCX) [file pcbi.1004690.s007.docx]

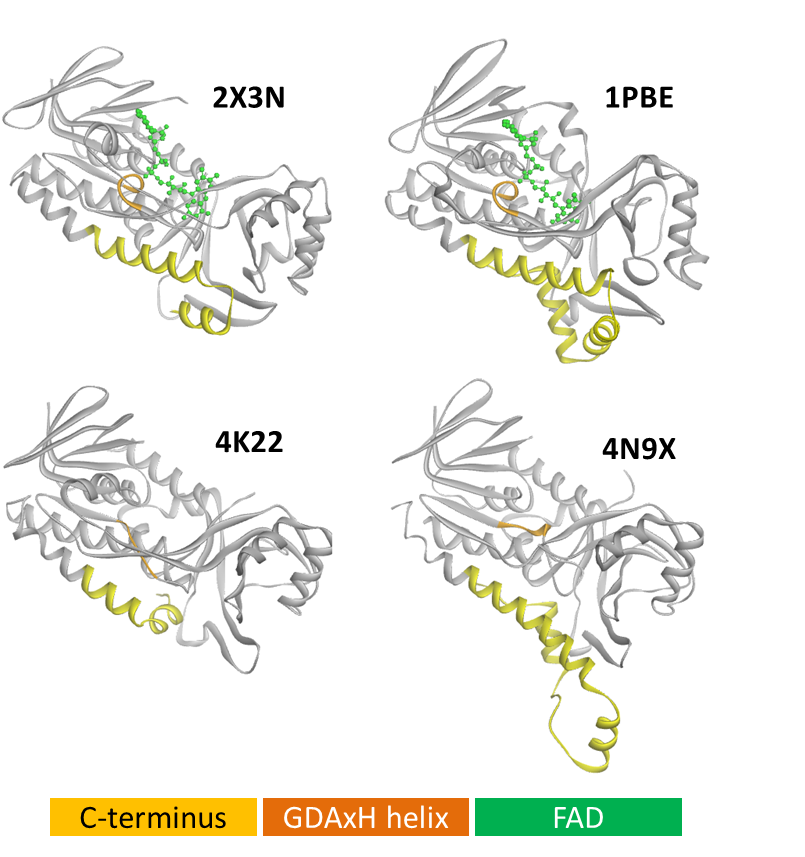


**S4 Fig.** **Structural comparison of the structural templates used in this work, 2X3N, 4K22, 4N9X, and 1PBE.** All four templates exhibit similar overall tertiary structures with a typical Rossmann-like β/α/β fold in addition to an FAD-binding domain. The C-terminus, which is missing in the 4K22 and 2X3N templates, exhibits an extended structure in 4N9X or a more compact one in 1PBE. It is noteworthy that the FAD co-factor is co-crystallized in 2X3N and 1PBE, making the latter valuable templates for a model of the FAD-dependent Coq6p enzyme. Main Chain RMSD WRT 1PBE: 2X3N (3.27 Å), 4N9X (5.45 Å), 4K22 (6.84 Å). Alpha carbon RMSD WRT 1PBE: 2X3N (3.32 Å), 4N9X (5.49 Å), 4K22 (6.83 Å).
