# Supplementary material for: Coenzyme Q Biosynthesis: Evidence for a Substrate Access Channel in the FAD-Dependent Monooxygenase Coq6
Source: PLoS Comput Biol. 2016 Jan 25;12(1):e1004690. doi: 10.1371/journal.pcbi.1004690 (PMC4726752; doi:10.1371/journal.pcbi.1004690)
Supplement: S5 Fig — Alignment was made with ConSurf and colored with the ClustalX colour scheme. The Coq6p insert consists of 51 residues (as identified in Fig 2) and is highlighted in the purple frame. The sequence of human Coq6 was manually added to the reference MSA. (DOCX) [file pcbi.1004690.s008.docx]

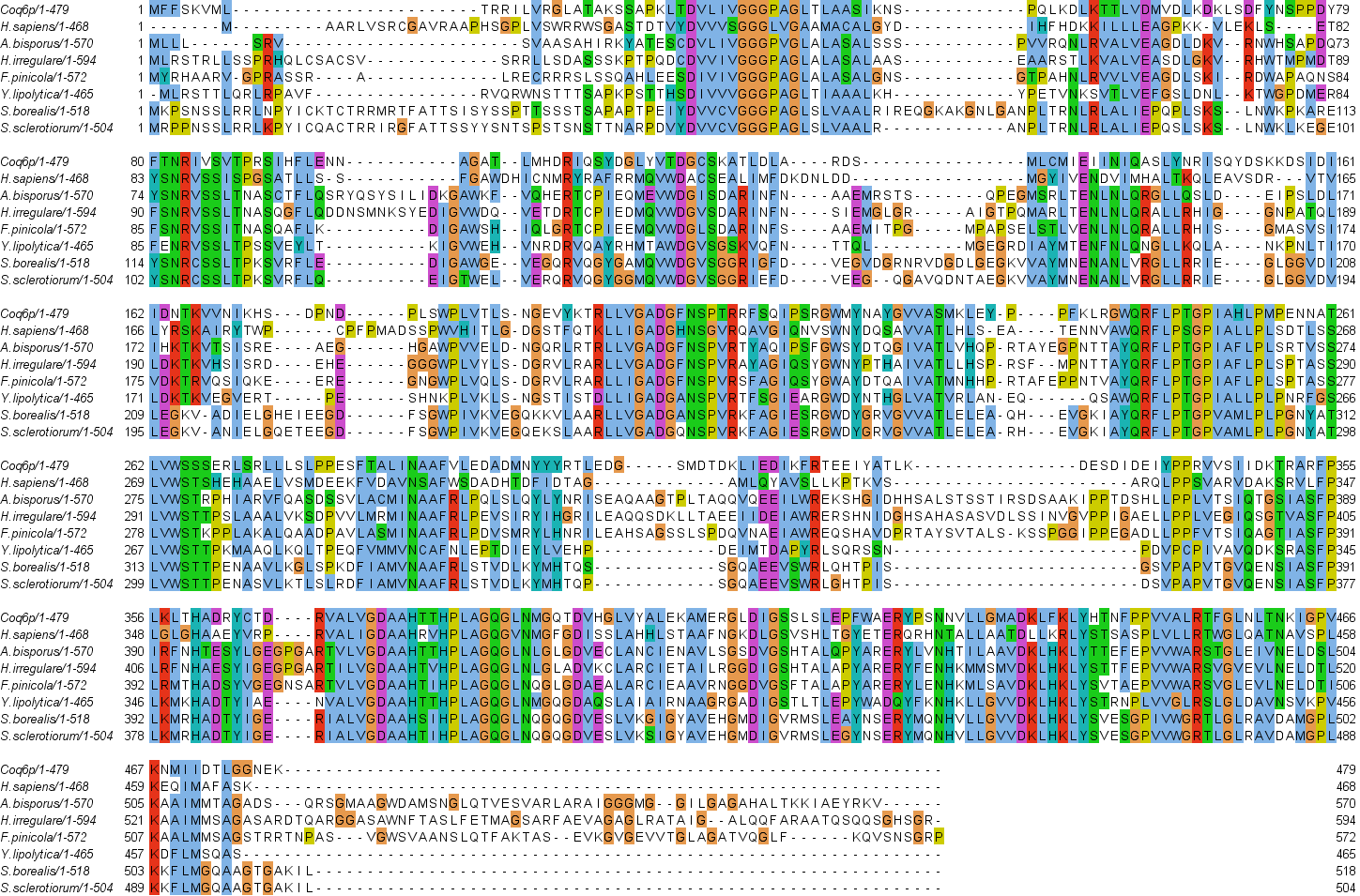


**S5 Fig.** **Alignment as extracted from the Multiple Sequence Alignment of Coq6p with 119 homologues as calculated by ConSurf.** Alignment was made with ConSurf and colored with the ClustalX colour scheme. The Coq6p insert consists of 51 residues (as identified in Fig 2) and is highlighted in the purple frame. The sequence of human Coq6 was manually added to the reference MSA.
