# Supplementary material for: Coenzyme Q Biosynthesis: Evidence for a Substrate Access Channel in the FAD-Dependent Monooxygenase Coq6
Source: PLoS Comput Biol. 2016 Jan 25;12(1):e1004690. doi: 10.1371/journal.pcbi.1004690 (PMC4726752; doi:10.1371/journal.pcbi.1004690)
Supplement: S6 Fig — A) Manually curated alignment used for the construction of Coq6p_MODELLER model. B) ROBETTA template construction alignment. C) Alignments used for the generation of the Coq6p homology models. I-TASSER template construction alignment. (DOCX) [file pcbi.1004690.s009.docx]

A


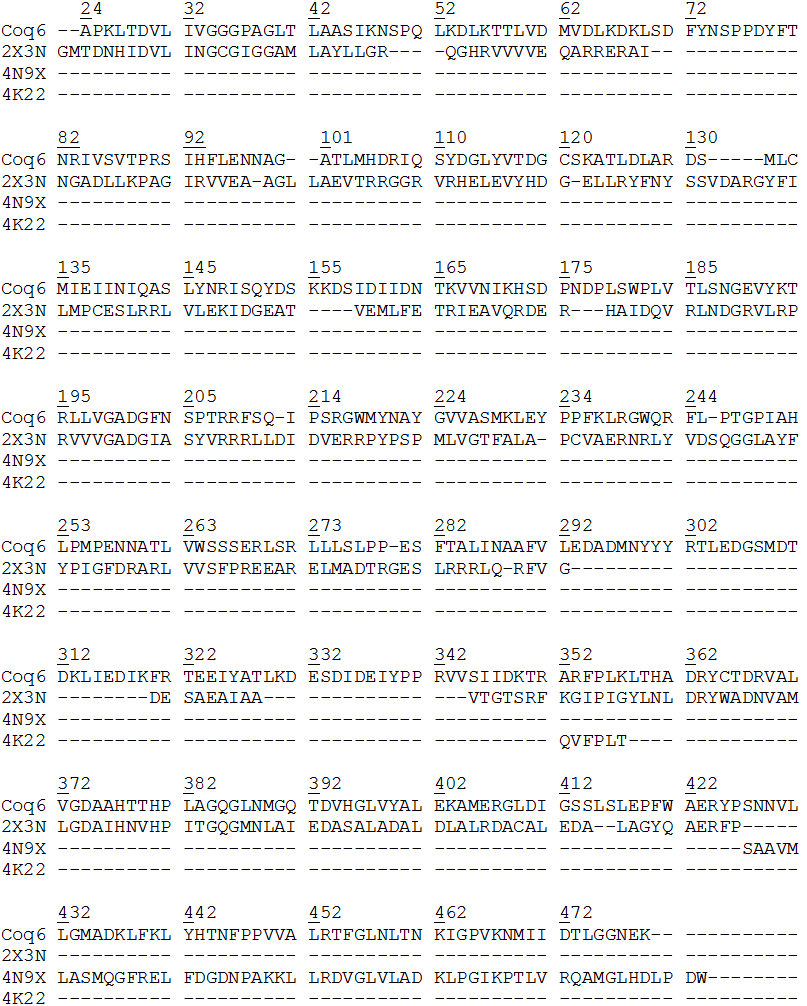


B


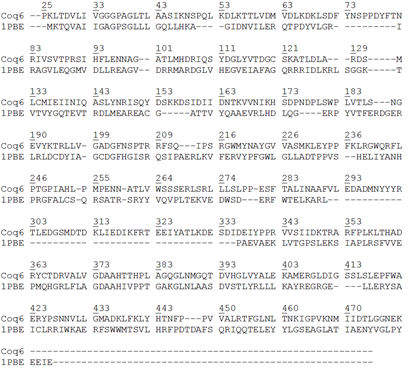


**S6 Fig A-B. Alignments used for the generation of the Coq6p homology models.** A) Manually curated alignment used for the construction of Coq6p_MODELLER model. B) ROBETTA template construction alignment.

C


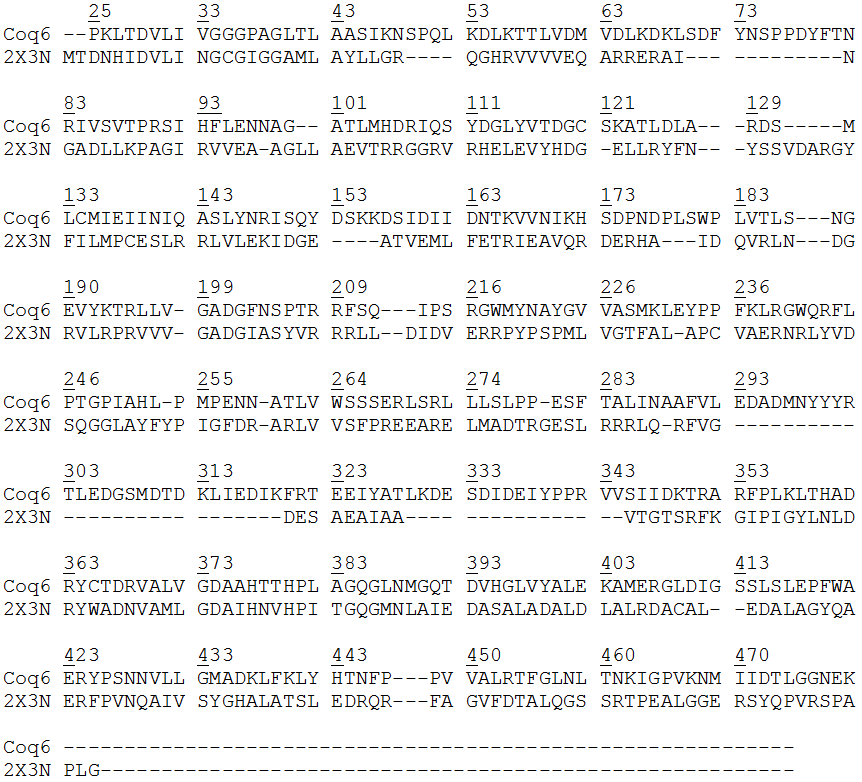


**S6 Fig C. Alignments used for the generation of the Coq6p homology models.** I-TASSER template construction alignment.
