# Supplementary material for: Coenzyme Q Biosynthesis: Evidence for a Substrate Access Channel in the FAD-Dependent Monooxygenase Coq6
Source: PLoS Comput Biol. 2016 Jan 25;12(1):e1004690. doi: 10.1371/journal.pcbi.1004690 (PMC4726752; doi:10.1371/journal.pcbi.1004690)
Supplement: S7 Fig — Red: Coq6p_ITASSER model; Green: Coq6p_ROBETTA model; Blue: Coq6p_MODELLER model. (DOCX) [file pcbi.1004690.s010.docx]

**
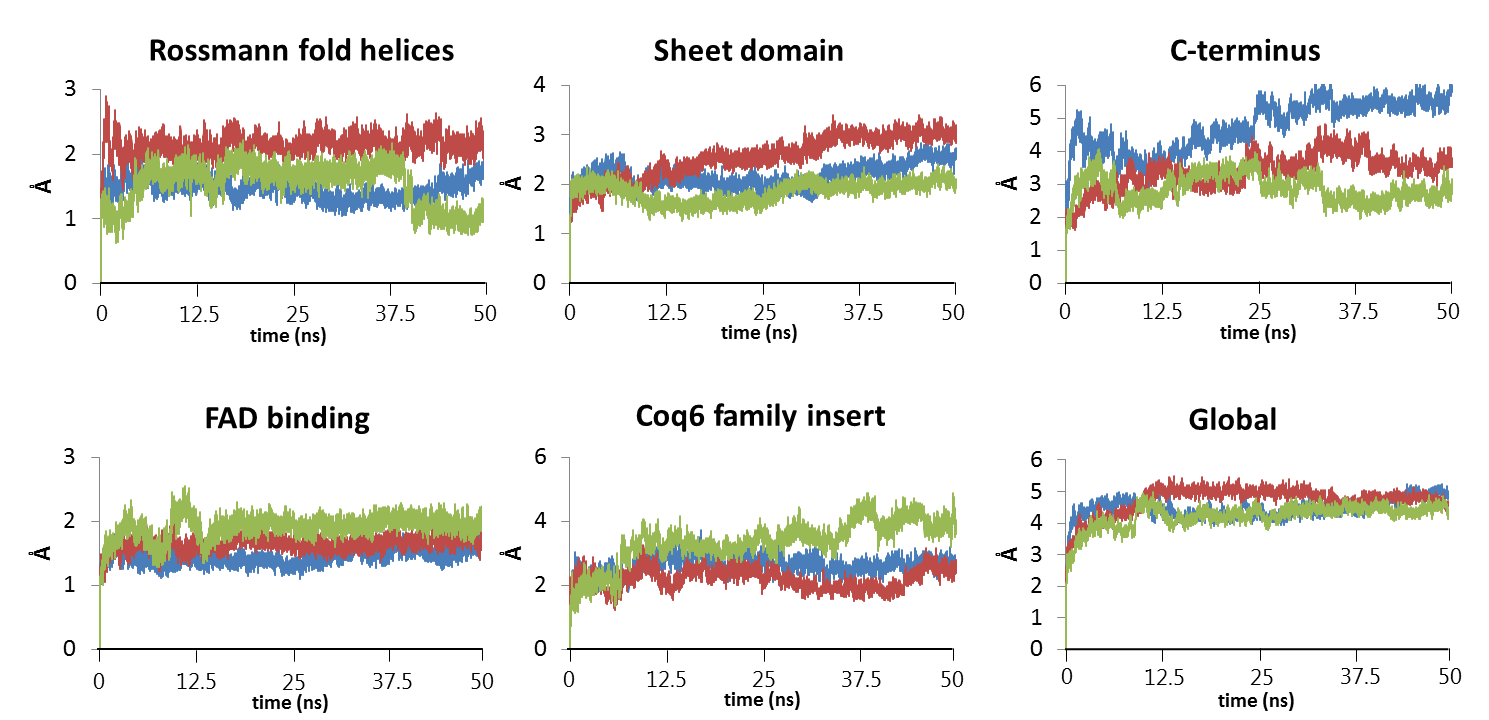
**

**S7 Fig.** **Time evolution over MD calculations of regional RMSDs for selected structural elements of Coq6p models.** Red: Coq6p_ITASSER model; Green: Coq6p_ROBETTA model; Blue: Coq6p_MODELLER model.
