# Supplementary material for: Coenzyme Q Biosynthesis: Evidence for a Substrate Access Channel in the FAD-Dependent Monooxygenase Coq6
Source: PLoS Comput Biol. 2016 Jan 25;12(1):e1004690. doi: 10.1371/journal.pcbi.1004690 (PMC4726752; doi:10.1371/journal.pcbi.1004690)
Supplement: S8 Fig — In the graphic, the β-sheet components turn (T) and extended conformation (E) are represented in teal and yellow respectively; isolated bridges are in dark yellow; degrees of helix are in pink (α-helix), blue (3–10 helix) and red (π-helix); random coils are in white. (DOCX) [file pcbi.1004690.s011.docx]

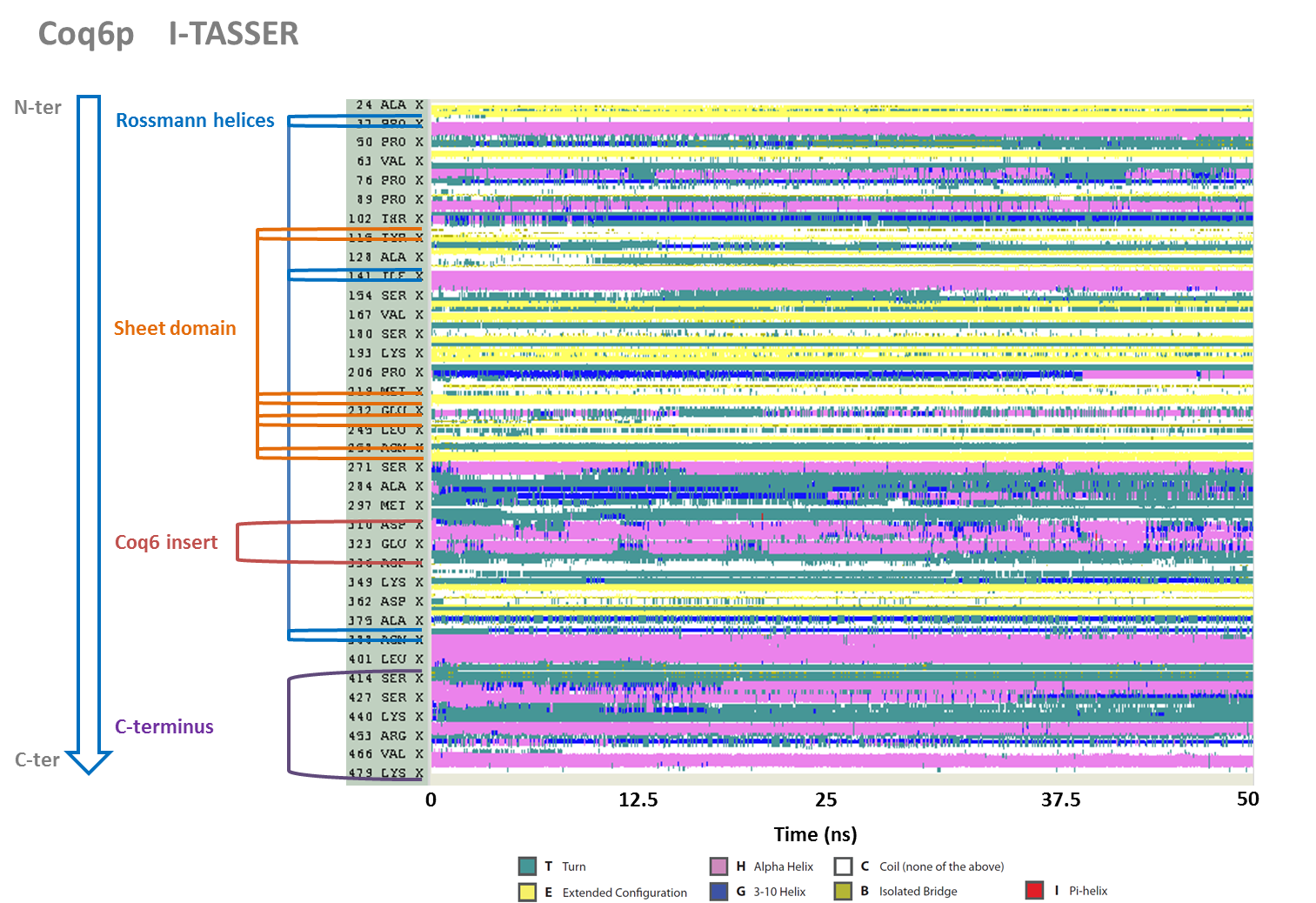


**S8 Fig. Secondary structure timeline analysis as computed by the timeline plugin contained in VMD for the Coq6p_I-TASSER model.** In the graphic, the β-sheet components turn (T) and extended conformation (E) are represented in teal and yellow respectively; isolated bridges are in dark yellow; degrees of helix are in pink (α-helix), blue (3-10 helix) and red (π-helix); random coils are in white.
