# Supplementary material for: Coenzyme Q Biosynthesis: Evidence for a Substrate Access Channel in the FAD-Dependent Monooxygenase Coq6
Source: PLoS Comput Biol. 2016 Jan 25;12(1):e1004690. doi: 10.1371/journal.pcbi.1004690 (PMC4726752; doi:10.1371/journal.pcbi.1004690)
Supplement: S11 Fig — Colors refer to helix 8 (yellow) and sub-sections of the Coq6p insert (N-terminal side of the insert in cyan, ascending part in red, C-terminal side of the insert in purple) as annotated in the alignment with 2X3N shown below. FAD is not shown for clarity. (DOCX) [file pcbi.1004690.s014.docx]

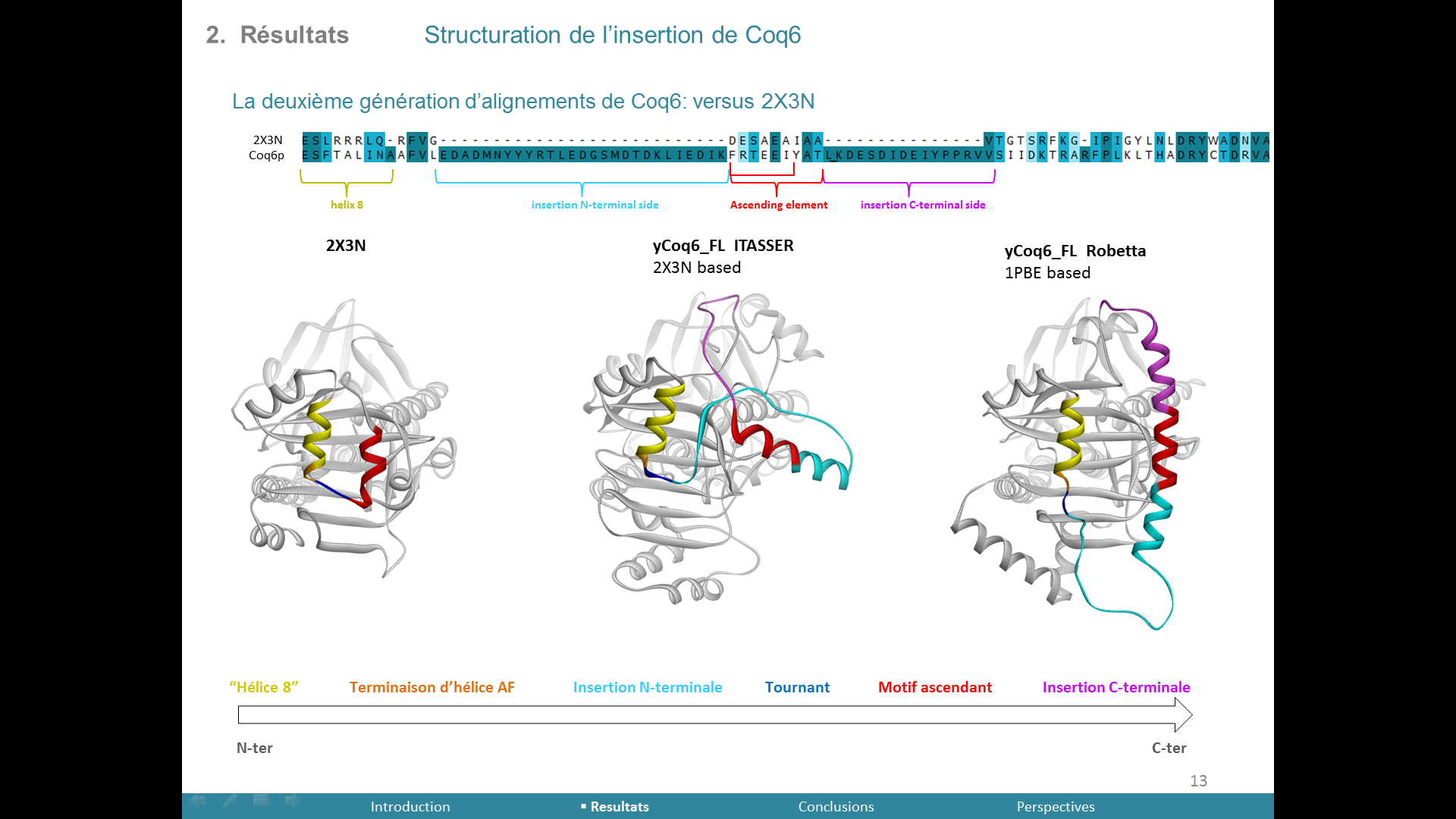


**helix8**

**insertion N-terminal side**

**ascending**

**element**

**insertion C-terminal side**


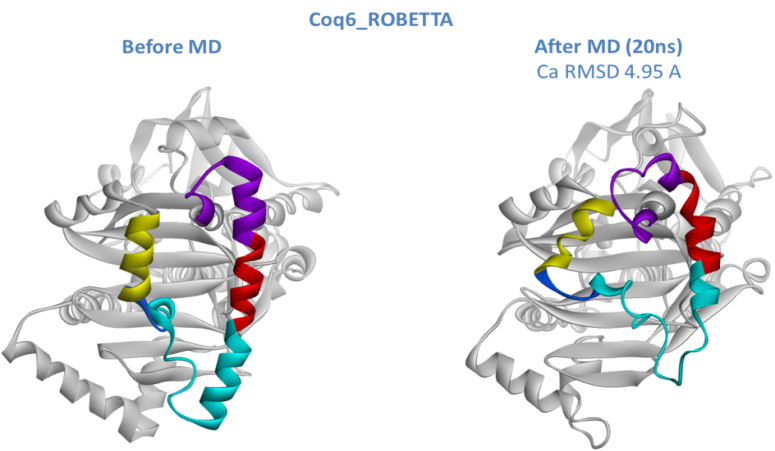

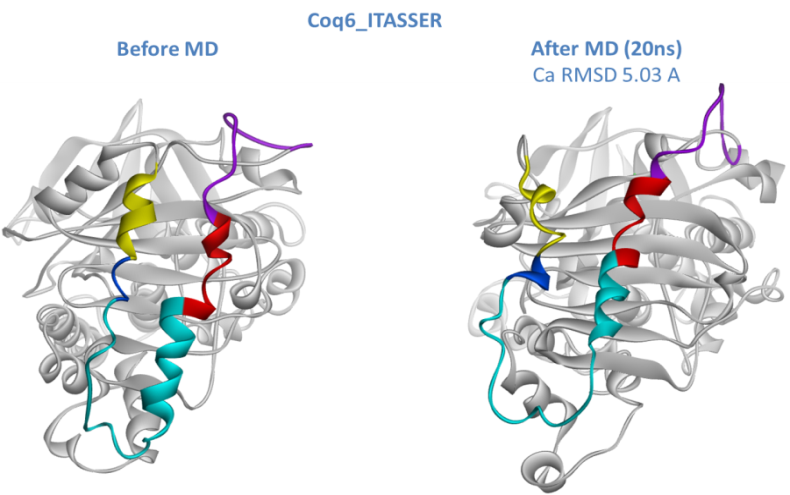

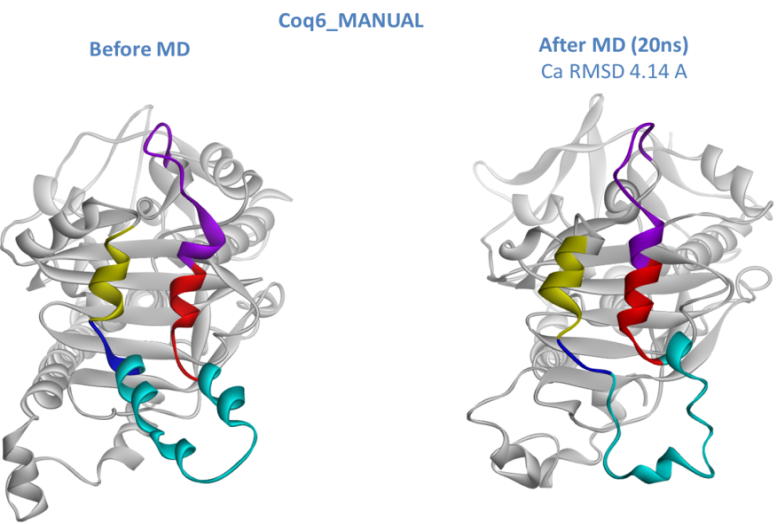


**Coq6_MODELLER**

**S11 Fig.** **Comparison of conformations of the three Coq6p models before and after 20 ns MD.** Colors refer to helix 8 (yellow) and sub-sections of the Coq6p insert (N-terminal side of the insert in cyan, ascending part in red, C-terminal side of the insert in purple) as annotated in the alignment with 2X3N shown below. FAD is not shown for clarity.
