# Supplementary material for: Coenzyme Q Biosynthesis: Evidence for a Substrate Access Channel in the FAD-Dependent Monooxygenase Coq6
Source: PLoS Comput Biol. 2016 Jan 25;12(1):e1004690. doi: 10.1371/journal.pcbi.1004690 (PMC4726752; doi:10.1371/journal.pcbi.1004690)
Supplement: S12 Fig — Top: tunnel 1; middle: tunnel 2; bottom: tunnel 3. Left: Coq6p_I-TASSER model; Middle: Coq6p_ROBETTA model; left: Coq6p_MODELLER model. (DOCX) [file pcbi.1004690.s015.docx]

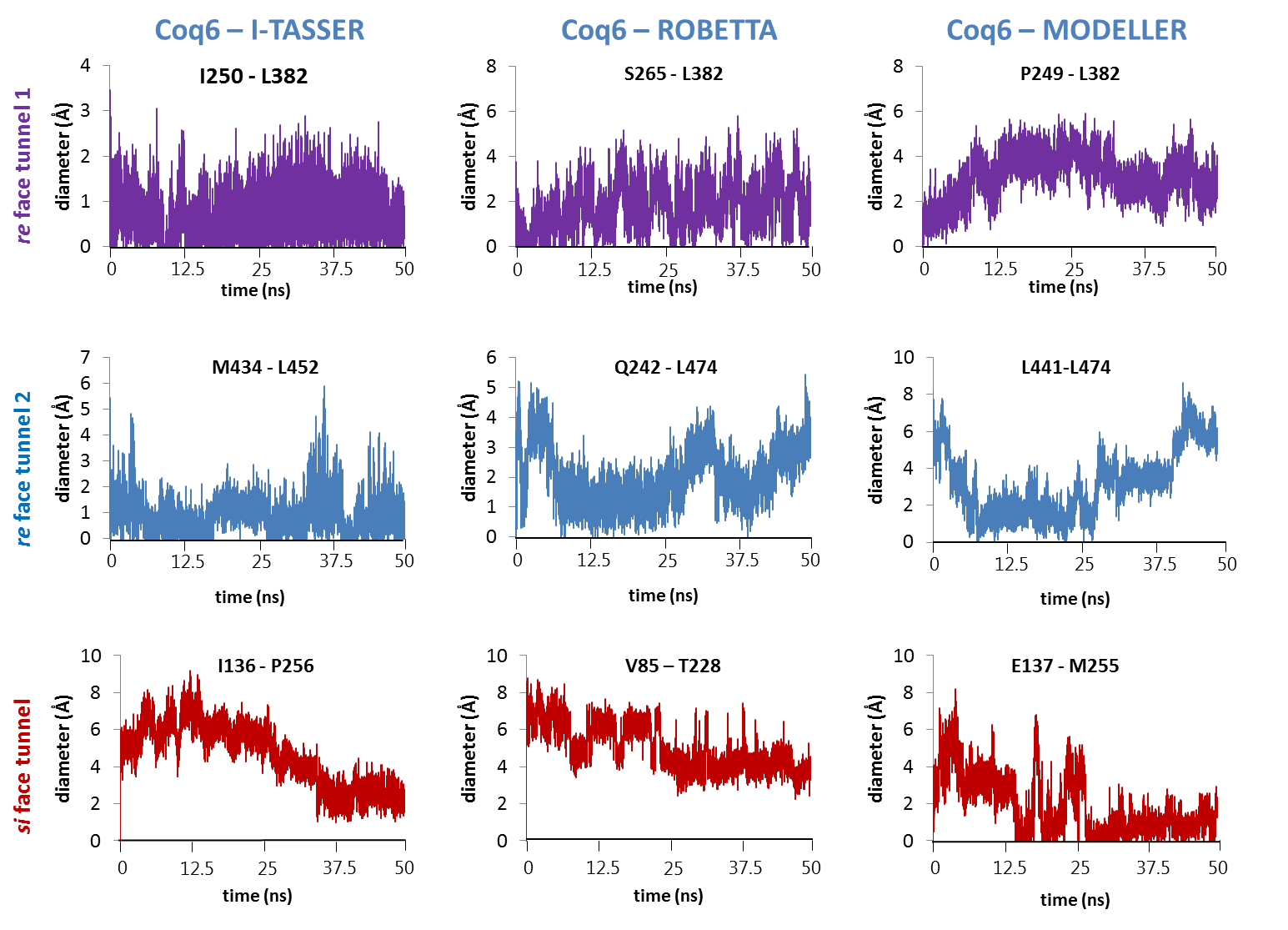


**S12 Fig.** **MD time-evolution in nanosecondes of the selected interatomic distances at choke points of tunnels 1, 2 and 3 in the three Coq6p models.** Top: tunnel 1; middle: tunnel 2; bottom: tunnel 3. Left: Coq6p_I-TASSER model; Middle: Coq6p_ROBETTA model; left: Coq6p_MODELLER model.
