# Supplementary material for: Coenzyme Q Biosynthesis: Evidence for a Substrate Access Channel in the FAD-Dependent Monooxygenase Coq6
Source: PLoS Comput Biol. 2016 Jan 25;12(1):e1004690. doi: 10.1371/journal.pcbi.1004690 (PMC4726752; doi:10.1371/journal.pcbi.1004690)
Supplement: S13 Fig — 4-hydroxybenzoic acid (4-HB) is prenylated by Coq2 to form 3-hexaprenyl-4-hydroxybenzoic acid (4-HB6). R represents the hexaprenyl tail on the biosynthetic products downstream of 4-HB6 and the numbering of the aromatic carbon atoms used in this study is shown on the reduced form of Q6, Q6H2. Upon inactivation of coq6 (path in red), 4-HB6 is decarboxylated (dashed arrow) and hydroxylated at position C1, yielding 3-hexaprenyl-4-hydroxyphenol (4-HP6). Vanillic acid (VA, in green) contains a methoxyl group on C5 and thus bypasses the C5-hydroxylation defect in coq6-deficient strains because it is converted into Q6 after its prenylation by Coq2 (see Ozeir et al. (2011) Chem Biol. 18, 1134–1142). (DOCX) [file pcbi.1004690.s016.docx]

**

**

**S13 Fig.** **S. cerevisiae Q_6_ biosynthetic pathway.** 4-hydroxybenzoic acid (4-HB) is prenylated by Coq2 to form 3-hexaprenyl-4-hydroxybenzoic acid (4-HB_6_). R represents the hexaprenyl tail on the biosynthetic products downstream of 4-HB_6_ and the numbering of the aromatic carbon atoms used in this study is shown on the reduced form of Q_6_, Q_6_H_2_. Upon inactivation of coq6 (path in red), 4-HB_6_ is decarboxylated (dashed arrow) and hydroxylated at position C1, yielding 3-hexaprenyl-4-hydroxyphenol (4-HP_6_). Vanillic acid (VA, in green) contains a methoxyl group on C5 and thus bypasses the C5-hydroxylation defect in coq6-deficient strains because it is converted into Q_6_ after its prenylation by Coq2 [1].
