# Supplementary material for: Coenzyme Q Biosynthesis: Evidence for a Substrate Access Channel in the FAD-Dependent Monooxygenase Coq6
Source: PLoS Comput Biol. 2016 Jan 25;12(1):e1004690. doi: 10.1371/journal.pcbi.1004690 (PMC4726752; doi:10.1371/journal.pcbi.1004690)
Supplement: S14 Fig — The enzyme conformation for the substrate docking calculations was selected with the active site geometric descriptor scoring-function derived from 1PBE (see Methods). The FAD co-factor is represented in green stick and the substrate in grey (aromatic head) and blue (isoprenyl chain). Several substrate poses are superimposed within the accessible volume (transparent pink) delimited by the re face substrate access tunnel 1. (DOCX) [file pcbi.1004690.s017.docx]

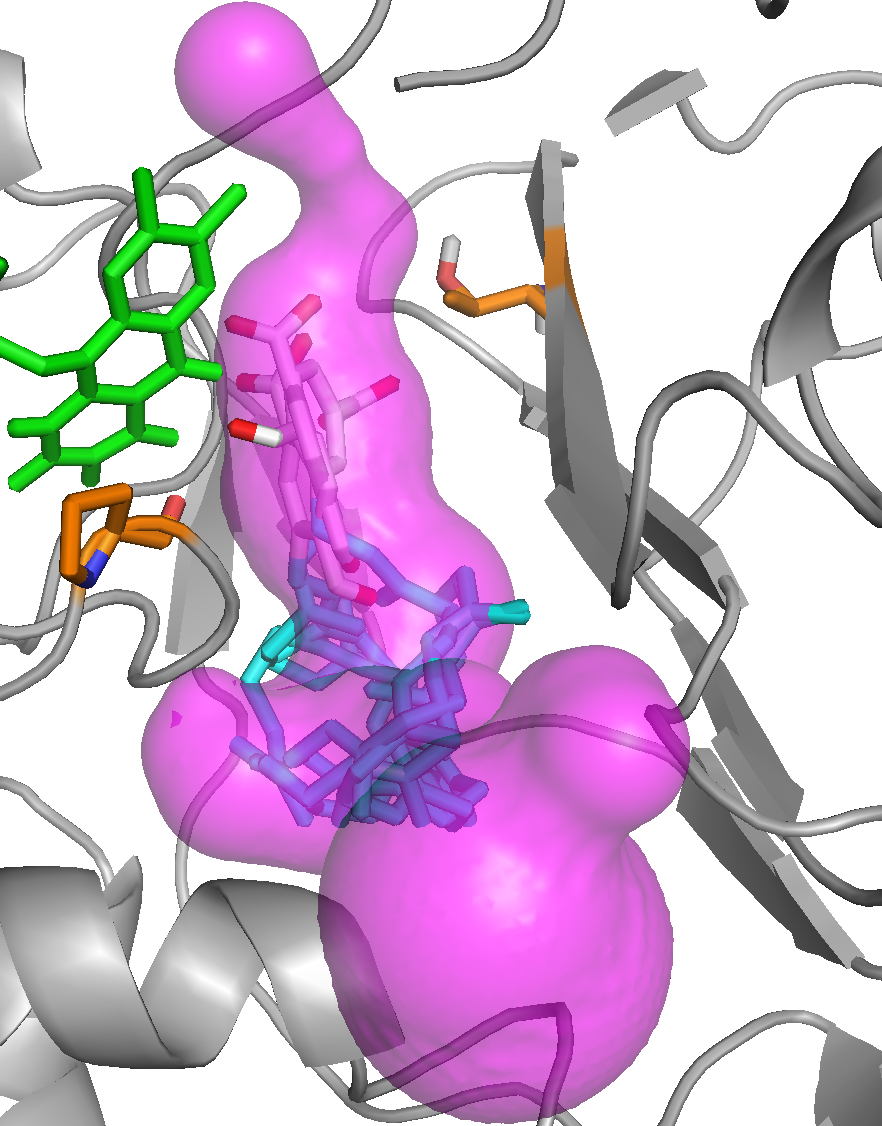


**T261**

**P381**


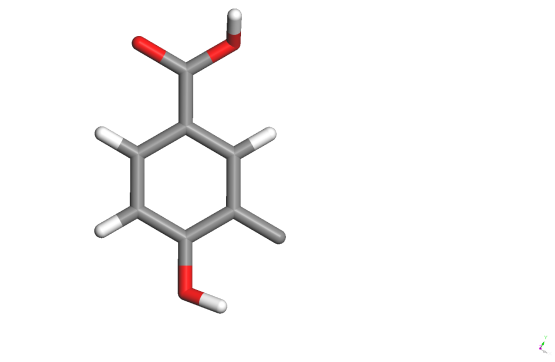


**S14 Fig.** **Docked poses of 3-hexaprenyl-4-hydroxyphenol model substrate (4-HB6) in Coq6p_MODELLER model.** The enzyme conformation for the substrate docking calculations was selected with the active site geometric descriptor scoring-function derived from 1PBE (see Methods). The FAD co-factor is represented in green stick and the substrate in grey (aromatic head) and blue (isoprenyl chain). Several substrate poses are superimposed within the accessible volume (transparent pink) delimited by the *re* face substrate access tunnel 1.
