# Supplementary material for: Coenzyme Q Biosynthesis: Evidence for a Substrate Access Channel in the FAD-Dependent Monooxygenase Coq6
Source: PLoS Comput Biol. 2016 Jan 25;12(1):e1004690. doi: 10.1371/journal.pcbi.1004690 (PMC4726752; doi:10.1371/journal.pcbi.1004690)
Supplement: S15 Fig — The hCoq6-Coq6p alignement based on the multiple sequence alignement of 119 Coq6p homologues was used for the identification of homologous residues, as follows: the mutations reported by Heeringa et al. (2011), R162X, G255R and A353D are identified as located at residues K155, G248, A361 in Coq6p. The homologous residue for D208H mutation reported for hCoq6 by Zhang et al. (2014) is D201 in Coq6p. The Y412C mutation in hCoq6 reported by Doimo et al. (2014) translates into Coq6p F420. (DOCX) [file pcbi.1004690.s018.docx]

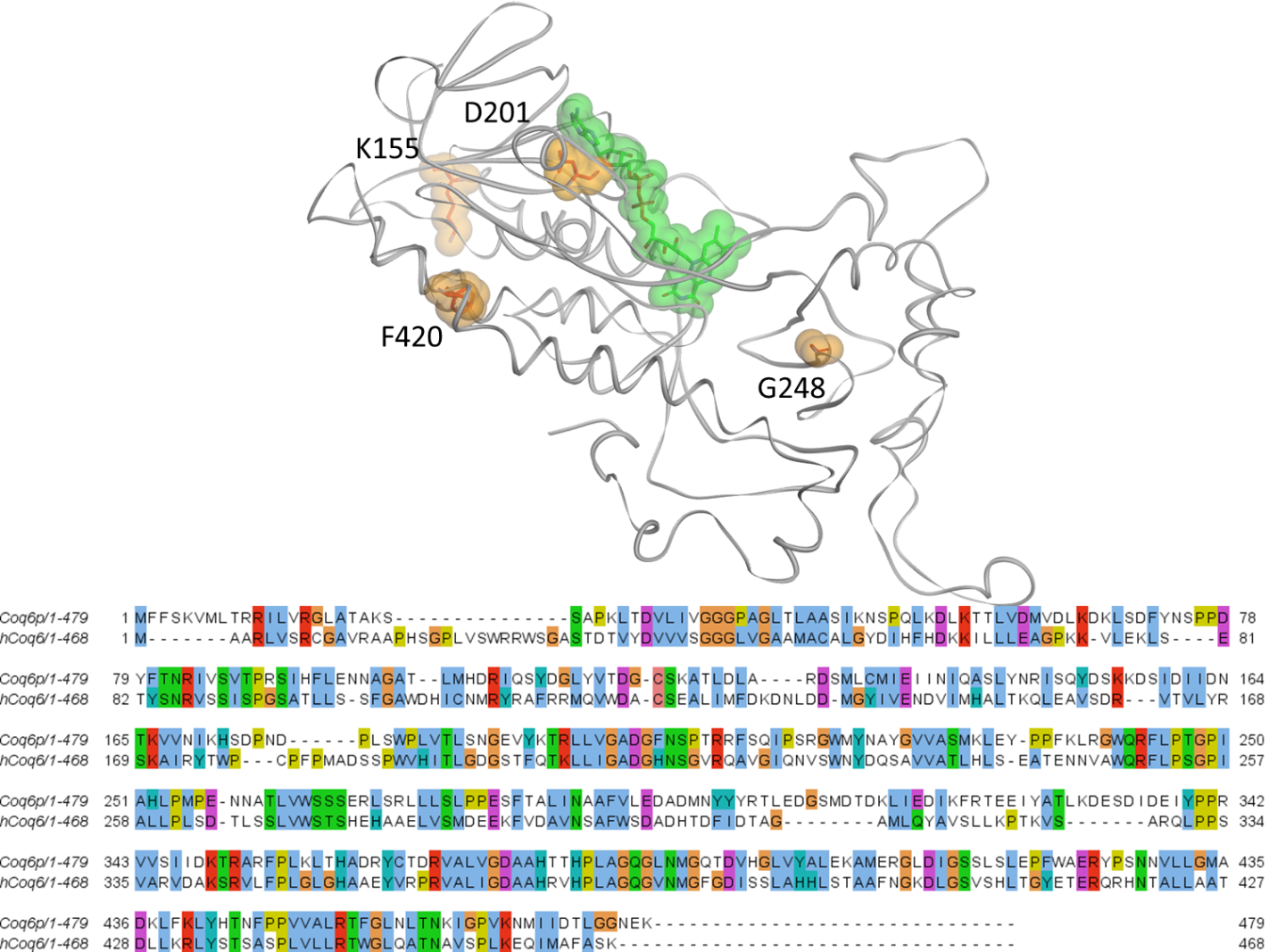


**S15 Fig.** **Schematic ribon representation of Coq6p with the positions of mutated residues reported in literature for hCoq6.** The hCoq6-Coq6p alignement based on the multiple sequence alignement of 119 Coq6p homologues was used for the identification of homologous residues, as follows: the mutations reported by Heeringa et al. (2011), R162X, G255R and A353D are identified as located at residues K155, G248, A361 in Coq6p. The homologous residue for D208H mutation reported for hCoq6 by Zhang et al. (2014) is D201 in Coq6p. The Y412C mutation in hCoq6 reported by Doimo et al. (2014) translates into Coq6p F420.
