# Supplementary material for: Coenzyme Q Biosynthesis: Evidence for a Substrate Access Channel in the FAD-Dependent Monooxygenase Coq6
Source: PLoS Comput Biol. 2016 Jan 25;12(1):e1004690. doi: 10.1371/journal.pcbi.1004690 (PMC4726752; doi:10.1371/journal.pcbi.1004690)
Supplement: S16 Fig — The FAD co-factor is represented in green sticks and the lipid/4HP6 in sticks within blue transparent volumes. In 4BJY the bound lipid enters through the large triangle formed by the C-terminus, with the lipid passing below Helix 12 (named re face channel 2 in the present work). In Coq6p, docking calculations and residue conservation suggest passage above Helix 12 (named re face channel 1 in the present work). The dashed circle in A highlights the position of the aromatic head of 4HP6. The dashed circle in B highlights the position of 3-Hydroxybenzoate as obtained in the co-crystallized 4BK1 crystal structure. (DOCX) [file pcbi.1004690.s019.docx]

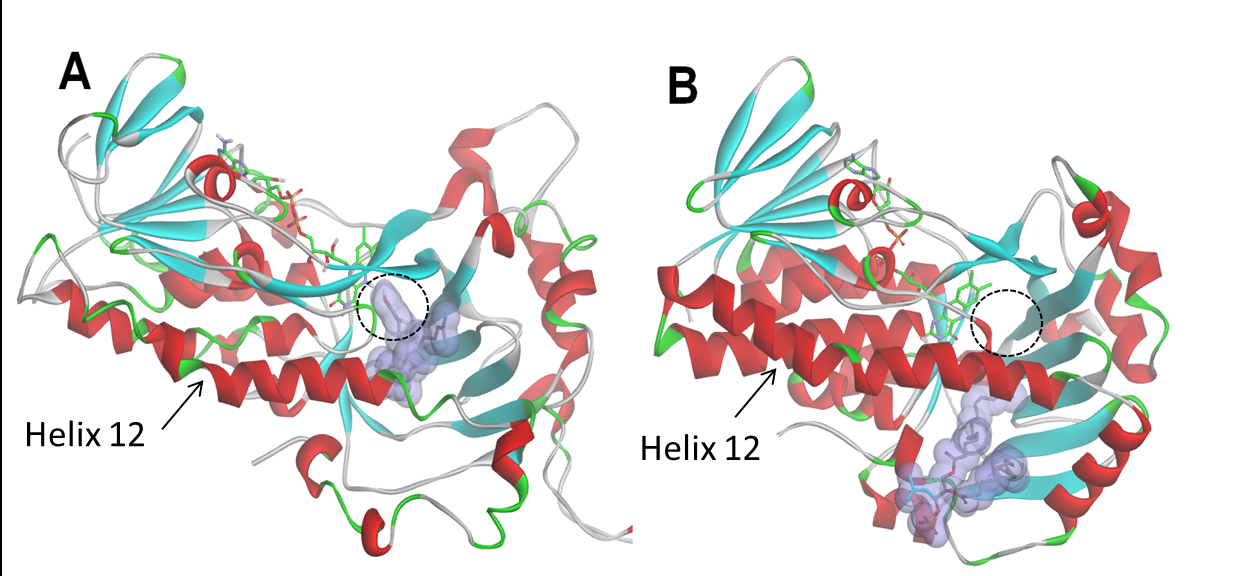


**S16 Fig. Comparison of the positioning of 4HP6 model substrate in Coq6p (A) and that of the bound lipid - a mixture of phosphatidylglycerol and phosphatidylethanol-amine- in 4BJY (B).** The FAD co-factor is represented in green sticks and the lipid/4HP6 in sticks within blue transparent volumes. In 4BJY the bound lipid enters through the large triangle formed by the C-terminus, with the lipid passing below Helix 12 (named re face channel 2 in the present work). In Coq6p, docking calculations and residue conservation suggest passage above Helix 12 (named *re* face channel 1 in the present work). The dashed circle in A highlights the position of the aromatic head of 4HP6. The dashed circle in B highlights the position of 3-Hydroxybenzoate as obtained in the co-crystallized 4BK1 crystal structure.
